# Synthesis of Catechol Derived Rosamine Dyes and Their Reactivity toward Biogenic Amines

**DOI:** 10.3390/molecules26165082

**Published:** 2021-08-22

**Authors:** Filipe Monteiro-Silva, Carla Queirós, Andreia Leite, María T. Rodríguez, María J. Rojo, Tomás Torroba, Rui C. Martins, Ana M. G. Silva, Maria Rangel

**Affiliations:** 1 Centre for Applied Photonics, INESC TEC, Faculdade de Ciências da Universidade do Porto, Rua do Campo Alegre, 4169-007 Porto, Portugal; filipe.m.silva@inesctec.pt (F.M.-S.); rui.c.martins@inesctec.pt (R.C.M.); 2 LAQV/REQUIMTE, Departamento de Química e Bioquímica, Faculdade de Ciências da Universidade do Porto, Rua do Campo Alegre, 4169-007 Porto, Portugal; carla.queiros@fc.up.pt (C.Q.); acleite@fc.up.pt (A.L.); 3 Departamento de Química, Facultad de Ciencias, Universidad de Burgos, 09001 Burgos, Spain; mtrod@ubu.es (M.T.R.); mjrocam@ubu.es (M.J.R.); ttorroba@ubu.es (T.T.); 4 LAQV/REQUIMTE, Instituto de Ciências Biomédicas de Abel Salazar, Rua Jorge de Viterbo Ferreira 228, 4050-313 Porto, Portugal; mrangel@icbas.up.pt

**Keywords:** microwave-assisted synthesis, catecholated rosamines, 9-aminopyronins, photophysical properties, biogenic amines detection

## Abstract

Functional organic dyes play a key role in many fields, namely in biotechnology and medical diagnosis. Herein, we report two novel 2,3- and 3,4-dihydroxyphenyl substituted rosamines (**3** and **4**, respectively) that were successfully synthesized through a microwave-assisted protocol. The best reaction yields were obtained for rosamine **4**, which also showed the most interesting photophysical properties, specially toward biogenic amines (BAs). Several amines including *n*- and *t*-butylamine, cadaverine, and putrescine cause spectral changes of **4**, in UV–Vis and fluorescence spectra, which are indicative of their potential application as an effective tool to detect amines in acetonitrile solutions. In the gas phase, the probe response is more expressive for spermine and putrescine. Additionally, we found that methanolic solutions of rosamine **4** and *n*-butylamine undergo a pink to yellow color change over time, which has been attributed to the formation of a new compound. The latter was isolated and identified as **5** (9−aminopyronin), whose solutions exhibit a remarkable increase in fluorescence intensity together with a shift toward more energetic wavelengths. Other 9-aminopyronins **6a**, **6b**, **7a**, and **7b** were obtained from methanolic solutions of **4** with putrescine and cadaverine, demonstrating the potential of this new xanthene entity to react with primary amines.

## 1. Introduction

Catechols are biologically reactive molecules that are widespread in nature and largely used in biomedicine and nanotechnology [1,2,3]. Catechol-functionalized molecules and materials are involved in many areas of chemistry and materials science [4], especially for producing: (i) metallopolymer networks for water purification [5]; (ii) adhesive functional hydrogels for biomedical applications [6]; and (iii) polymers with antimicrobial properties [7]. Catechols can be easily oxidized via autoxidation in the presence of molecular oxygen, photooxidation, or through the addition of chemical agents or enzymatic oxidants [8,9,10]. *o*-Quinones are the most common oxidation products of catechols, being reactive intermediates in several reactions. They can also be attacked by nucleophiles such as amines, whose attack is dependent of the electron withdrawing or donating nature of the substituents present in the quinone skeleton [11], solvent, and pH.

Amines, on the other hand, are produced on the ton scale worldwide and are used in pharmaceutical, agricultural, and several industry-based applications [12]. Biogenic amines (BAs) are nitrogen-based compounds originating mainly from the decarboxylation of amino acids present in animals, microorganisms, and plants [13]. Amines can also display significant reactivities and their radical cations play a significant role in biological systems such as in the enzymatic metabolism of endogenous amines to the corresponding imines [14]. The detection of amines is a relevant research area with impact on the diagnosis of several diseases as well as industrial and environmental monitoring, besides food quality control. In this context, fluorescent probes have emerged as a rapid, simple, and accurate molecular tool to detect trace amounts of BAs. Optical detection of BAs is mainly based on: (i) the use of functional organic dyes such as phenanthridines [15], naphthalenes [16], benzothiazoles [17], porphyrinoids [18,19]; (ii) supramolecular hydrogels [20]; and (iii) boron complexes [21,22]. Recently revised by A. Gupta, the aggregation-induced emission (AIE) phenomenon by different materials presents itself as a method with high potential for the detection of amines by an increase or a quench of the fluorescence process [23].

**Figure 1:**
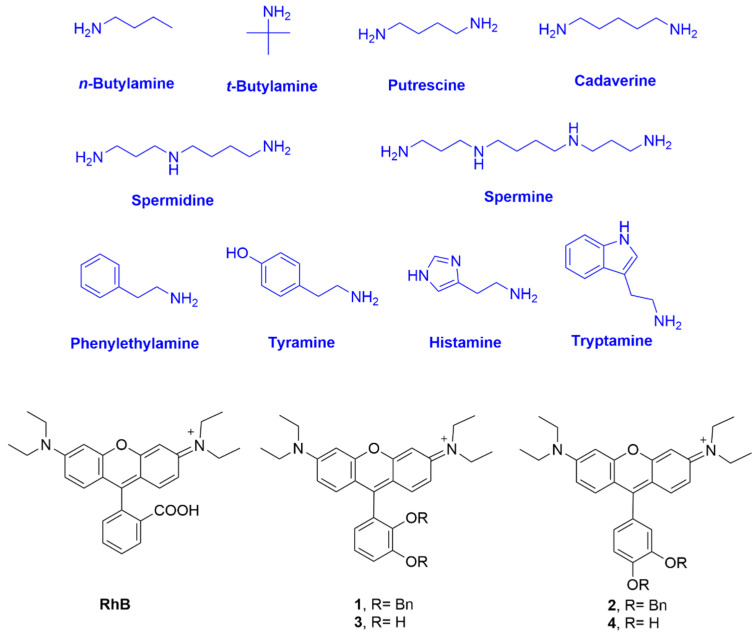
Chemical structures of the biogenic amines used in the studies, **RhB** and rosamines **1**–**4**.

In recent years, our group has synthesized several fluorescein and rhodamine-catechol conjugates and studied their interaction with transition metal ions from the coordination point of view, also exploring their ability to act as metal ion sensors [24,25]. Inspired by the protonation and redox behavior of catechols and by the excellent photophysical properties of Rhodamine B (**RhB**) and other xanthene derivatives [26], we present herein a simple design strategy to synthesize rosamines **1**–**4**, with the purpose of detecting BAs by the direct conjugation of 2,3- and 3,4-dihydroxyphenyl rings with the xanthene platform (Figure 1).

## 2. Results and Discussion

**Scheme 1:**
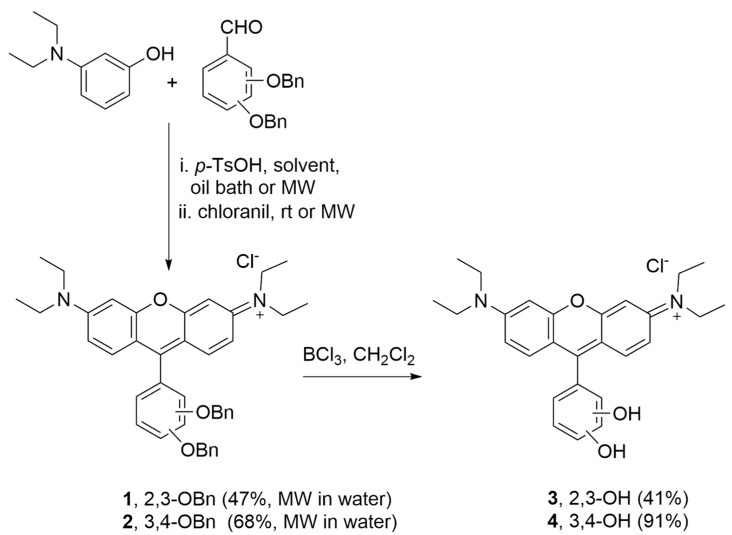
Synthetic route to obtain rosamines **1**–**4**.

The synthetic route for rosamines **1**–**4** is simple and effective (Scheme 1). It consists of the condensation of the appropriate dibenzyloxybenzaldehyde and 3-(diethylamino)phenol in the presence of a catalytic amount of *p*-toluenesulfonic acid (*p*-TsOH), with subsequent oxidation with chloranil to give **1** and **2**. The removal of the benzyl protecting groups was performed using boron trichloride in dichloromethane to afford **3** and **4**. After screening different experimental conditions for condensation including (i) the heating method (oil bath or microwave irradiation, MW) and (ii) the reaction solvent (propionic acid or water), we verified that the combination of MW and water provided higher yields for compounds **1** and **2** (47% and 68%, respectively) in a remarkable shorter period of time (10 min) (please refer to the Materials and Methods section for more details).

Rosamines (**1**–**4**) were characterized by ^1^H NMR, ^13^C NMR, HRMS, UV–Vis, and fluorescence spectroscopy. These were obtained as a dark pink powder soluble in organic solvents, but were only sparingly soluble in an aqueous solution. Their photophysical properties including absorption extinction coefficients (*ε*) and fluorescence quantum yield (*Φ*_F_) were studied in aprotic and protic solvents including chloroform, acetonitrile, and methanol and compared with **RhB**. The results are listed in Table 1.

The data showed that the molecular rigidity and the electron density of the substituent groups have remarkable effects on the photophysical properties of the compounds, namely: (i) in chloroform, the absorption bands of the catecholated compounds were in the shorter wavelength region than those of the benzylated ones; (ii) a marginally higher *Φ*_F_ value (0.60) was obtained for **1** in chloroform, where the orthogonal OBn substituent constrains the rotation of the 9-phenyl ring and thus enhances its fluorescence properties [27]; and (iii) the presence of the catechol substituent (**3** and **4**) remarkably decreased the *Φ*_F_ value of the molecules due to the intramolecular photoinduced electron transfer (PET), which occurs from the hydroxyl electron donating groups (D) to the xanthene electron acceptor fragment (A), producing the donor–acceptor (D–A) pair [28]. Although the catecholate derivatives are always less emissive than the respective benzylated derivatives, the emission of **3** and **4** (especially in terms of *Φ*_F_ values) was significantly higher in acetonitrile than in chloroform and methanol. Lower Stokes shifts were observed for rosamines **1** and **2** in chloroform, the same being true for **RhB**. On the other hand, for rosamines **3** and **4**, the solvent did not influence the Stokes shift values as significantly.

Taking into account the yields obtained in the synthesis and the peculiar spectral properties of the catechol derived compounds, we considered rosamine **4** as the most interesting dye in this series. Accordingly, we started by studying the influence of pH variation (2 < pH < 12) in the fluorescence intensity of rosamine **4**. The graphical representation of the fluorescence intensity as a function of the pH value showed a decrease in intensity from 2 to approximately 10 (please refer to Figure S27 in the Supplementary Materials). In this range, two deprotonations can be observed both related to the deprotonation of the two hydroxyl groups, the first occurs for pH values from pH 2 to 6.5 (zwitterionic form) and the second deprotonation occurs for pH values above 6.5 until pH 9.90 (anionic form).

Subsequently, we investigated the color and fluorescence qualitative changes of acetonitrile solutions of **4** in the presence of the most common BAs diluted in water at room temperature using 2, 4, and 8 equiv. of amine. As shown in Figure S28 of the Supplementary Materials, by adding increasing amounts of amines, there was a change (quench of fluorescence) observed under ultraviolet radiation, indicating that rosamine **4** is sensitive to 2 equiv. of cadaverine, putrescine, spermidine, and spermine. Furthermore, upon addition of 4 and 8 equiv., a quench in fluorescence was also observed for *n*-butylamine and histamine, respectively.

**Figure 2:**
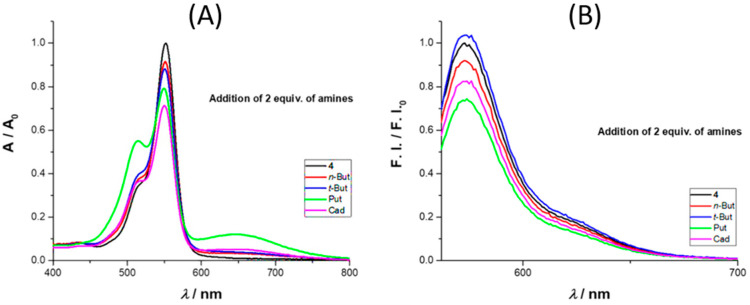
Comparison of the absorbance (**A**) and emission intensity (**B**) spectra of **4** toward 2 equiv. of *n*-butylamine (*n*-But, red line), *t*-butylamine (*t*-But, blue line), putrescine (Put, green line), and cadaverine (Cad, magenta line) in solution (acetonitrile, 25 °C).

We also tested the influence of selected amines, namely *n*- and *t*-butylamine, cadaverine, and putrescine on the spectroscopic behavior of rosamine **4** in acetonitrile, using UV–Vis and fluorescence (Figure 2). With the addition of 2 equiv. of amine, there was an absorbance decrease at 552 nm, more expressive for cadaverine. On the emission spectra, there was an intensity quench at 573 nm with the addition of 2 equiv. of amine, with the exception of *t*-butylamine, where a small increase was observed. In terms of percentage values, for the maximum absorbance, the decrease was in the order of 8%, 12%, 21%, and 29% for *n*-butylamine, *t*-butylamine, putrescine, and cadaverine, respectively, while for the emission intensity, the percentage values were 8%, 4% (increase), 27%, and 18%, respectively.

**Figure 3:**
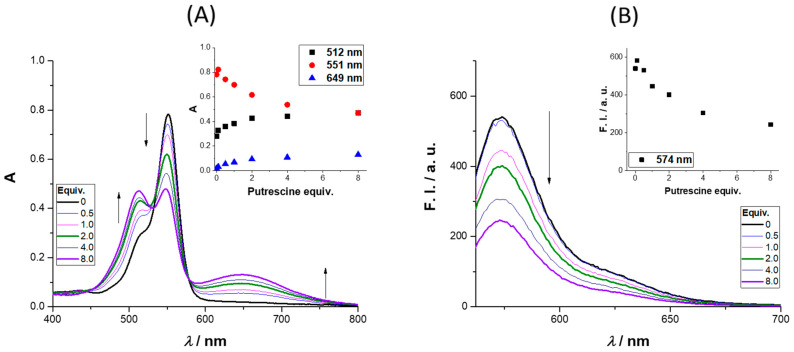
Absorption (**A**) and emission (**B**) spectra of **4** upon the addition (0–8 equiv.) of putrescine in CH_3_CN (the inset graphs represent the variation in absorbance (left) and fluorescence intensity (right) for the wavelengths that display more intensity variation (512, 551, and 649 nm; 574 nm, respectively). |4| = 35 μM and *λ*_exc_ = 551 nm.

The absorbance and emission spectra of **4** after the addition of up to 8 equiv. of putrescine is presented in Figure 3 (further amine results are available in the Supplementary Materials). The absorbance spectra revealed three regions of interest, two with an intensity increase (512 and 649 nm) and the third (at 551 nm) where a quench was observed as the molar equivalents of putrescine increased. The intensity enhancement regions can be used to monitor the presence of putrescine in the concentration range (0 to 122 μM). The region between 649 and 657 nm is particularly interesting due to the enhancement of the absorbance; the same behavior was observed for cadaverine (please refer to the Supplementary Materials, Figure S29, and for different concentrations of cadaverine (0.1–40 equiv.) please refer to Figures S30 and S31). The excitation of **4** in the presence of 8 equiv. of putrescine at 649 nm did not lead to a new emission band, the same being true for the other BAs. Analyzing the emission spectra, a quench percentage of 55% was obtained for an addition of 8 equiv. of putrescine, much higher values than those observed for *n*-butylamine and cadaverine (12% and 32%, respectively), whereas for *t*-butylamine, a slight increase was obtained (6%).

**Figure 4:**
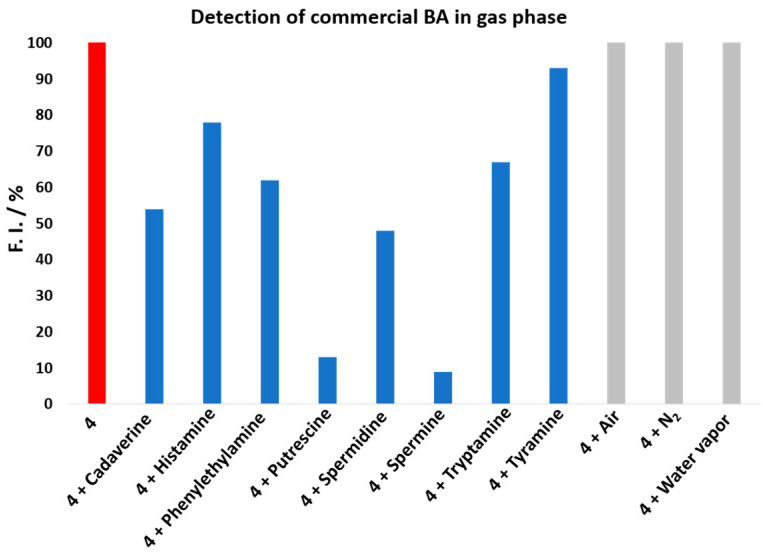
Comparison of the fluorescence intensity change of **4** toward BA in the gas phase at *λ*_exc_ 533 nm. Probe **4** in red, Probe **4** + BA in blue, and Probe **4** + Controls (air, N_2_ and water vapor) in grey.

To demonstrate that rosamine **4** can be used in the detection of volatile amine vapors, which are highly toxic, irritant, and corrosive, being important indicators of food quality, for example, in the assessment for fish freshness [29], we investigated its reactivity toward BAs in the gas phase. The study was carried out by injecting vapors of different commercial BAs in solutions of **4** dissolved in acetonitrile (see the Materials and Methods section for more details and the apparatus in Figure S32 in the Supplementary Materials). The spectrophotometrical measurements were carried at 533 nm. As shown in Figure 4, nitrogen, air, and water vapor did not affect rosamine **4**, since the value of fluorescence intensity did not change when they were injected. However, when the commercial BA vapor was introduced, a decrease in the fluorescence intensity was observed, which was more expressive upon spermine addition, resulting in decreases greater than 90%, 87% for putrescine, 52% for spermidine, 47% for cadaverine, 32% for tryptamine, 21% for histamine, and 8% for tyramine. The results for putrescine and cadaverine confirmed rosamine **4** sensitivity toward these BAs with higher quenching percentages in the gas phase comparatively to those reported in solution for 8 equiv.

**Figure 5:**
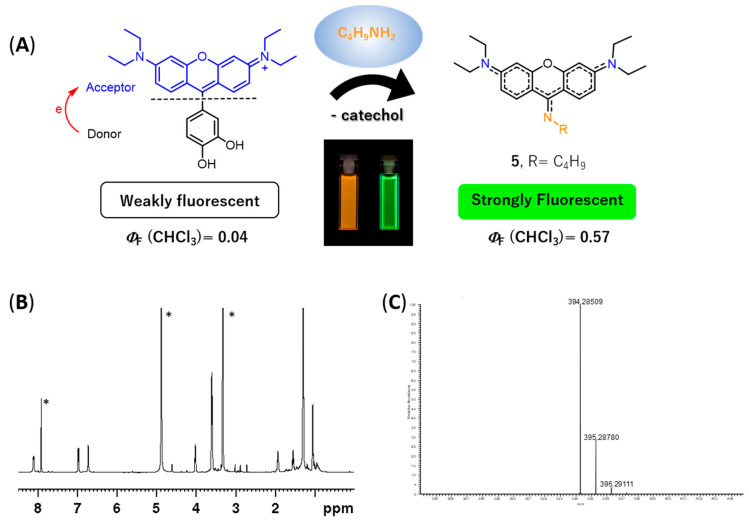
Reactivity of rosamine **4** with *n*-butylamine. (**A**) Scheme of the reaction of rosamine **4** with *n*-butylamine leading to the formation of 9-aminopyronin **5**. Rosamine **4** itself is weakly fluorescent due to the intramolecular PET quenching (left hand-side cuvette). However, after reaction with *n*-butylamine and chromatographic purification, 9-aminopyronin **5** can be isolated, which exhibits strong fluorescence (right hand-side cuvette); (**B**) ^1^H NMR spectrum of **5** in methanol-d_4_, * denote solvent peaks (chloroform and methanol); (**C**) ESI-MS spectrum of **5**.

During the experimental assays, when compound **4** was brought to the presence of amines, we observed that—particularly for methanolic solutions of **4** with *n*-butylamine—a color change from pink to yellow was observed over time, at room temperature and at 4 °C (refrigerator), which appeared to be due to the formation of a new compound. To verify the aforementioned hypothesis, a solution of rosamine **4** and *n*-butylamine (15 equiv.) in methanol was prepared, which was kept at 4 °C for 120 h. The TLC analysis of the resulting solution revealed the presence of a new compound with a bright yellow color, which was subsequently isolated by preparative thin layer chromatography and characterized by NMR and ESI-MS. The corresponding ^1^H NMR spectrum (please refer to Figure 5B and Figure S33 of the Supplementary Materials) exhibited: (i) three set of signals from 6.73 to 8.12 ppm due to the resonance of the xanthene ring (no signals due to the catechol unit were detected indicating the absence of the catechol substituent in the molecule) and (ii) a new set of signals appeared at 1.05, 1.55, 1.93, and 4.02 ppm due to the entry of a butylamine moiety, suggesting the formation of 9-aminopyronin **5** (Figure 5A). A comparison of the ^1^H NMR spectra of compounds **4** and **5** is provided in the Supplementary Materials (please refer to Figure S35). Further evidences of **5** were obtained by ESI-MS, through the molecular ion peak [M + H]^+^ at *m/z* 394.2851 (Figure 5C and Figure S39 in the Supplementary Materials).

In fact, 9-aminopyronin dyes are described in the literature as chemically stable dyes having high molar absorption coefficients and high fluorescence quantum yields. Being initially introduced by K. Burgess et al. [30], their synthesis typically involves the reaction of xanthenone derivatives with amines in the presence of trifluoromethanesulfonic anhydride or oxalyl chloride [31,32,33,34]. Alternatively, 9-aminopyronins can be prepared by reacting 9H-xanthene-9-thione with electron-withdrawing groups such as 4-aminopyridine and 2-aminoquinoline [35]. However, to the best of our knowledge, there are no preceding reports describing the synthesis of 9-aminopyronine dyes from catecholate xanthenes with amines.

Several attempts have been conducted in order to improve the yield of aminopyronin **5**, which included reactions at 4 °C using chloroform or methanol and the use of MW heating (*N*-methylpyrrolidine as solvent, 200 °C, 10 min; detailed procedures in the Materials and Methods section). The best result was obtained when methanol was used as a solvent (4 °C, for ca. 144 h) with a 53% yield.

**Scheme 2:**
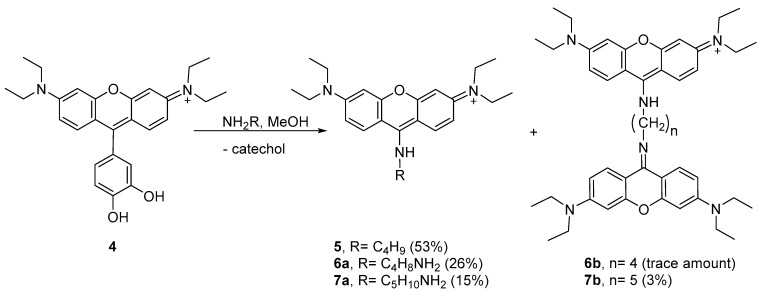
Reaction involving the nucleophilic attack of alkylamines to **4**, leading to aminopyronin **5**, **6a**, **6b**, **7a** and **7b**.

Considering the results obtained with *n*-butylamine, we decided to study the peculiar reactivity of **4** using two other nucleophilic amines—putrescine and cadaverine. Using methanol as the solvent at 4 °C, the reactions took place in different extensions, leading to aminopyronins **6a,b** and **7a,b** (Scheme 2, see ^1^H NMR spectra in Figures S36–S38 in the Supplementary Materials).

The reaction of **4** with putrescine mainly afforded the monomer **6a**, as supported by the NMR spectral analysis and MALDI-TOF mass spectrometry, showing [M + H]^+^ at *m/z* 409.409 (theoretical [M + H]^+^ = 409.296), while only a trace amount of dimer **6b** was obtained. Higher reaction extension was achieved when cadaverine was used, affording monomer **7a** together with the dimer **7b**. The ^1^H NMR profile of **7a** follows the same profile as compound **5** while the MALDI-TOF spectrum showed the molecular ion [M + H]^+^ at *m/z* 423.450 (theoretical [M + H]^+^ = 423.312) supporting the structure **7a**. In the ^1^H NMR spectrum of dimer **7b**, the presence of two equivalent xanthene rings per one cadaverine frame was confirmed by the 12 aromatic protons and the 10 aliphatic protons, which is consistent with the MALDI-TOF spectrum that showed [M + H]^+^ at *m/z* 743.969 (theoretical [M + H]^+^ = 743.501).

For comparison purposes, the photophysical properties of all isolated aminopyronins (**5**, **6a**, **7a**, **7b**) were accessed in chloroform, methanol, and acetonitrile (Table 2). All compounds presented an intense yellow color (naked eye), exhibiting absorption in the range of 430–440 nm with high extinction coefficients (10.6 × 10^4^–4.35 × 10^4^). Their emissions were in the range of 507–526 nm with large Stokes shifts (72–88 nm; 3265–3820 cm^−1^), values around 5.4–6.6 times higher than those found for rosamines **1**–**4**. High fluorescence quantum yields were obtained, especially in chloroform (*Φ*_F_ = 0.57, 0.36, 0.58, and 0.53, respectively, for **5**, **6a**, **7a**, and **7b**), a fact in clear contrast with the starting rosamines **3** and **4** for which low *Φ*_F_ values were obtained in chloroform (Table 1).

Considering the possible similar reactivity of 2,3-dihydroxylphenyl substituted rosamine **3**, a similar study was performed from a solution of **3** and *n*-butylamine (15 equiv.) in methanol at 4 °C for 120 h. In this case, TLC analysis of the resulting solution showed a complex mixture of products very difficult to purify by chromatographic methods. It is very probable that, according to the generally accepted crosslinking chemistry of catechols and amines [11], the catechol unit would suffer different nucleophilic attacks of *n*-butylamine by either Michael-type addition or via the formation of a Schiff base, thus supporting the formation of many different products. However, in the case of the reaction of rosamine **4** with *n*-butylamine, 9-aminopyronin **5** was obtained as the major product of the reaction. The data suggest that the nucleophilic attack of the amine to the electrophilic 9-position of the xanthene takes place with the formation of the addition adduct, and that the loss of the catechol moiety occurs, potentially, during the isolation process.

## 3. Materials and Methods

Reagents and solvents were purchased as reagent-grade and used without further purification unless otherwise stated. NMR spectra were recorded on a Bruker Avance III 400 spectrometer (Bruker BioSpin GmbH, Rheinstetten, Germany; 400.15 MHz for ^1^H and 100.63 MHz for ^13^C) or on a Bruker Avance III HD 600 spectrometer (Bruker BioSpin GmbH, Rheinstetten, Germany; 600.13 MHz for ^1^H and 150.92 MHz for ^13^C). Chemical shifts (δ) were reported in ppm and coupling constants (*J*) in Hz; internal standard was TMS. Two-dimensional ^1^H/^1^H correlation spectra (COSY), gradient selected ^1^H/^13^C heteronuclear single quantum coherence (HSQC), and ^1^H/^13^C heteronuclear multiple bond coherence (HMBC) spectra were acquired using the standard Bruker software. Flash chromatography was carried out using silica gel (230–400 mesh,Merck, Kenilworth, NJ, USA). Microwave-assisted reactions were carried out in a CEM Discovery Labmate circular single-mode cavity instrument (300 W max magnetron power output) from CEM Corporation (CEM Microwave Technology Ltd., Buckingham, UK). Reactions were performed under closed-vessel conditions. MS analysis of compounds **1**, **2**, and **5** were carried out by electrospray ionization (ESI) in a LTQ-Orbitrap-XL instrument (Thermo Fischer Scientific, Winsford, UK) with the following ESI source parameters: electrospray needle voltage 3.1 kV, sheath gas nitrogen 6, capillary temperature 275 °C, capillary voltage 21 V, and tube lens voltage 55 V. Ionization polarity was adjusted according to sample. Mass spectra of rosamines **3** and **4** were acquired by Unidade De Espectrometria De Masas of Santiago de Compostela and microanalyses were acquired by Unidad De Análisis Elemental of Santiago de Compostela. Mass spectra of compounds **6a** and **7a,b** were acquired on a Bruker UltrafleXtreme MALDI-TOF/TOF equipped with a 200 Hz smartbeam laser (Bruker Daltonik GmbH, Bremen, Germany). Electronic absorption spectra were recorded with a Varian Cary bio50 spectrophotometer (Agilent Technologies, Santa Clara, CA, USA), equipped with a Varian Cary single-cell Peltier accessory, using 1 cm path-length quartz cells. Steady-state fluorescence measurements were carried out in a Varian spectrofluorometer, model Cary Eclipse (Agilent Technologies, Santa Clara, CA, USA), equipped with a constant-temperature cell holder (Peltier single-cell holder) with 5 nm slit width for excitation and emission. All photophysical assays were performed under controlled temperature conditions (25 °C), using the maximum *λ*_exc_ and the appropriate *λ*_em_ range, for each rosamine and considering the different solvents used. Rosamine stock solutions were prepared in anhydrous dimethylsulfoxide (DMSO) and DMSO percentage was kept under 1% regarding the reported studies with different solvents. Rhodamine B (**RhB**) was used as standard in the photophysical assays and the respective solutions were prepared in anhydrous ethanol. Solutions of the compounds, in appropriate concentration ranges, were prepared previously to molar absorptivity coefficients (*ε*) and fluorescence quantum yield (*Φ*_F_) determination assays. Generally, fluorescence quantum yield determination was done combining the literature-described [36,37], pH variation study was carried out from a stock solution of rosamine **4** in water, and dilutions were performed with ionic force adjustment (0.1 mol/L NaCl). Each pH adjustment from 2–12 was obtained by the addition of small amounts of concentrated HCl and NaOH, followed by UV–Vis and fluorescence measurements between 200–800 nm or 555–700 nm for UV–Vis and fluorescence, respectively.

Synthesis of benzylated precursors: To a solution of 2,3-dihydroxybenzaldehyde or 3,4-dihydroxybenzaldehyde (1.00 g, 7.24 mmol) and K_2_CO_3_ (2.20 g, 15.9 mmol) in DMF (20 mL) at 0 °C, benzyl bromide (1.89 mL, 15.9 mmol) was added drop-by-drop, under N_2_ atmosphere. After stirring at 0 °C for 15 min, the reaction mixture was allowed to warm up to room temperature and the stirring was maintained for 4 h. After that time, the reaction mixture was precipitated into an ice/water mixture and neutralized using citric acid. The white solid obtained was filtered, washed with water, dissolved in chloroform, and crystallized in *n*-hexane to afford the respective products.

2,3-Dibenzyloxybenzaldehyde: Yield 75% (1.73 g). ^1^H NMR (400 MHz, CDCl_3_) δ: 5.19 and 5.20 (4H, 2s, 2xCH_2_C_6_H_5_), 7.12 (1H, dt, *J* 8.0 Hz and *J* 0.6 Hz, H-Ar), 7.23–7.26, 7.32–7.43 and 7.47–7.49 (12H, 3m, H-Ar), 10.26 (1H, s, CHO). ^13^C NMR (CDCl_3_, 100.62 MHz) δ: 71.3 (CH_2_), 76.5 (CH_2_), 119.6, 119.9, 124.2, 127.6, 128.3, 128.5, 128.6, 128.7, 128.8, 130.5, 136.3, 151.5, 152.1, 190.2 (CHO). MS (EI) *m/z*: 318 M^+^. C_21_H_18_O_3_ ¼ H_2_O calcd. C 78.12, H 5.78; found C 78.46, H 5.34.

3,4-Dibenzyloxybenzaldehyde: Yield 84% (1.94 g). ^1^H NMR (400 MHz, CDCl_3_) δ: 5.22 and 5.26 (4H, 2s, 2×CH_2_C_6_H_5_), 7.02 (1H, d, *J* 8.0 Hz, H-5), 7.32–7.49 (12H, m, H-Ar), 9.81 (1H, s, CHO) ppm. HRMS (ESI) *m/z*: calcd. for C_21_H_19_O_3_^+^, 319.129; found, 319.133.

### 3.1. Synthesis of Rosamines ***1*** and ***2***

(a) Via conventional heating using propionic acid as solvent: a solution of 3-(diethylamino)phenol (0.28 g, 1.72 mmol) with the appropriate benzaldehyde (0.27 g, 0.86 mmol) and *p*-TsOH (17.2 mg, 0.10 mmol) in propionic acid (10 mL) was placed in a reaction flask and heated to 65 °C for 16 h. After cooling to room temperature, the mixture was neutralized with NaOAc (3 mol/L, 100 mL). The resulting suspension was extracted with chloroform. The combined organic extracts were dried (Na_2_SO_4_ anhydrous) and the solvent evaporated. The resulting residue was dissolved in 40 mL of a mixture of CHCl_3_/MeOH (1:1), to which chloranil (0.10 g, 0.43 mmol) was added. The mixture was vigorously stirred for 2 h and concentrated in vacuum. The residue was purified by flash chromatography using a mixture of CHCl_3_/MeOH (9:1) and vacuum dried.

(b) Via MW using propionic acid or water as solvent: a solution of 3-(diethylamino)phenol (0.14 g, 0.86 mmol) with the appropriate benzaldehyde (0.13, 0.43 mmol) and *p*-TsOH (10.0 mg, 0.06 mmol) in water or propionic acid (5 mL) was placed in a 10 mL reaction vial, which was then sealed and placed in the cavity of a CEM microwave reactor. The reaction was irradiated at 80 °C (1 min ramp to 80 °C and 10 min hold at 80 °C, using 100 W maximum power). The solvent was decanted and the resulting solid was dissolved in 10 mL of a mixture of CHCl_3_/MeOH (1:1), to which chloranil (0.10 g, 0.43 mmol) was added. The mixture was placed into the cavity of the CEM microwave using 1 min ramp to 60 °C and 10 min hold at 60 °C, using 50 W maximum power. The residue was purified by flash chromatography using a mixture of CHCl_3_/MeOH (9:1) and vacuum dried.

Rosamine **1**: (a) Yield 36% (95 mg); (b) Yields 43% (113 mg) and 47% (124 mg) in propionic acid and water, respectively. ^1^H NMR (400 MHz, CDCl_3_) δ: 1.33 (12H, t, *J* 7.2 Hz, 4×CH_3_), 3.64 (8H, q, *J* 7.2 Hz, 4×CH_2_), 4.88 (2H, s, 2′-CH_2_C_6_H_5_), 5.27 (2H, s, 3′-CH_2_C_6_H_5_), 6.74–6.76 (3H, m, H-Ar), 6.77 (2H, d, *J* 2.4 Hz, H-4 and H-5), 6.82 (2H, dd, *J* 9.4 and *J* 2.4 Hz, H-2 and H-7), 6.93–7.03 (3H, m, H-Ar), 7.19 (2H, d, *J* 9.4 Hz, H-1 and H-8), 7.23–7.27 and 7.36–7.52 (7H, 2m, H-Ar) ppm. ^13^C NMR (100 MHz, CDCl_3_) δ: 12.7 (CH_3_), 46.2 (CH_2_), 71.2 (3′-CH_2_C_6_H_5_), 75.2 (2′-CH_2_C_6_H_5_), 96.2 (C-4 and C-5), 113.7, 114.0, 116.0 (C-2 and C-7), 122.4, 124.8, 126.7, 127.6, 127.8, 128.0, 128.4, 128.7, 132.2 (C-1 and C-8), 136.2, 136.6, 145.5 (C-2′), 152.3 (C-3′), 155.1 (C-9), 155.4 (C-3 and C-6), 157.7 (C-4a and C-5a) ppm. HRMS (ESI) *m/z*: calcd. for C_41_H_43_N_2_O_3_^+^, 611.327; found 611.326. 

Rosamine **2**: (a) Yield 12% (32 mg); (b) Yields 61% (160 mg) and 68% (179 mg) in propionic acid and water, respectively. ^1^H NMR (400 MHz, CDCl_3_) δ: 1.32 (12H, t, *J* 7.2 Hz, 4×CH_3_), 3.63 (8H, q, *J* 7.2 Hz, 4×CH_2_), 5.24 (2H, s, 3′-CH_2_C_6_H^5^), 5.31 (2H, s, 4′-CH_2_C_6_H_5_), 6.77 (2H, d, *J* 2.4 Hz, H-4 and H-5), 6.80 (2H, dd, *J* 9.6 and *J* 2.4 Hz, H-2 and H-7), 6.89 (1H, d, *J* 2.0 Hz, H-2′), 6.92 (1H, dd, *J* 8.0 and *J* 2.0 Hz, H-6′), 7.17 (1H, d, *J* 8.0 Hz, H-5′), 7.27 (2H, d, *J* 9.6 Hz, H-1 and H-8), 7.31–7.45 (8H, m, H-Ar), 7.53 (2H, d, *J* 7.2 Hz, H-Ar) ppm. ^13^C NMR (100 MHz, CDCl_3_) δ: 12.8 (CH_3_), 46.2 (CH_2_), 71.2 and 71.3 (CH_2_C_6_H_5_), 96.5 (C-4 and C-5), 113.2 (C-1a and C-8a), 114.1 (C-2 and C-7), 114.4 (C-5′), 116.9 (C-2′), 123.8 (C-6′), 124.2 (C-1′), 127.37, 127.41, 128.0, 128.2, 128.76, 128.79, 132.2 (C-1 and C-8), 136.6, 136.7, 148.4 (C-3′), 151.1 (C-4′), 155.4 (C-3 and C-6), 157.1 (C-9), 158.0 (C-4a and C-5a) ppm. HRMS (ESI) *m/z*: calcd. for C_41_H_43_N_2_O_3_^+^, 611.327; found, 611.326.

### 3.2. Synthesis of Rosamines ***3*** and ***4***

A 1 mol/L solution of boron trichloride in dichloromethane (2 mL) was dropped slowly into an ice-bath cooled suspension of rosamine **1** or **2** (85.6 mg, 0.14 mmol) in dry dichloromethane (8 mL) under a N_2_ atmosphere. The mixture was stirred at room temperature for 18 h. Methanol (20 mL) was added to stop the reaction, followed by several washings with acetone and methanol. After removal of the solvent in vacuum, the residue was precipitated with methanol/acetone.

Rosamine **3**: Yield 41% (25 mg). ^1^H NMR (400 MHz, DMSO-d_6_) δ: 1.21 (12H, t, *J* 7.2 Hz, 4×CH_3_), 3.65 (8H, q, *J* 6.8 Hz, 4×CH_2_), 6.66 (1H, d, *J* 7.6 Hz, H-6′), 6.89 (1H, dd, *J* 8.0 and *J* 7.6 Hz, H-5′), 6.96 (2H, d, *J* 2.2 Hz, H-4 and H-5), 7.07 (1H, d, *J* 8.0 Hz, H-4′), 7.14 (2H, dd, *J* 9.4 and *J* 2.2 Hz, H-2 and H-7), 7.26 (2H, d, *J* 9.4 Hz, H-1 and H-8), 9.00 (1H, s, OH), 9.96 (1H, s, OH) ppm. ^13^C NMR (100 MHz, DMSO-d_6_) δ: 12.5 (CH_3_), 45.3 (CH_2_), 95.8 (C-4 and C-5), 113.0 (C-1a and C-8a), 114.3 (C-2 and C-7), 117.2 (C-4′), 119.3 (C-1′), 119.5 (C-5′), 120.4 (C-6′), 131.9 (C-1 and C-8), 143.0 (C-2′), 145.8 (C-3′), 155.1 (C-3 and C-6), 155.6 (C-9), 157.4 (C-4a and C-5a) ppm. MS (ESI) *m/z*: 431.2 M^+^. C_27_H_31_ClN_2_O_3_.3/2CH_2_Cl_2_ calcd. C, 57.59, H, 5.77, N, 4.71; found C, 58.07, H, 5.46, N, 4.75. 

Rosamine **4**: Yield 91% (55 mg). ^1^H NMR (400 MHz, DMSO-d_6_) δ: 1.22 (12H, t, *J* 7.2 Hz, 4×CH_3_), 3.65 (8H, q, *J* 6.8 Hz, 4×CH_2_), 6.79 (1H, dd, *J* 8.0 and *J* 2.1 Hz, H-6′), 6.94 (3H, d, *J* 2.1 Hz, H-4, H-5 and H-2′), 7.05 (1H, d, *J* 8.0 Hz, H-5′), 7.16 (2H, dd, *J* 9.8 and *J* 2.1 Hz, H-2 and H-7), 7.47 (2H, d, *J* 9.8 Hz, H-1 and H-8), 9.77 (2H, s broad, 2×OH) ppm. ^13^C NMR (100 MHz, DMSO-d_6_) δ: 12.5 (CH_3_), 45.3 (CH_2_), 95.9 (C-4 and C-5), 112.5 (C-1a and C-8a), 114.2 (C-2 and C-7), 116.0 (C-5′), 117.3 (C-2′), 121.7 (C-6′), 122.3 (C-1′), 132.1 (C-1 and C-8), 145.6 (C-3′), 147.8 (C-4′), 154.9 (C-3 and C-6), 157.3 (C-9), 157.4 (C-4a and C-5a) ppm. MS (ESI) *m/z*: 431.2 M^+^. C_27_H_31_ClN_2_O_3_.½CH_2_Cl_2_ calcd. C, 64.83, H, 6.33, N, 5.50; found C, 64.41, H, 6.28, N, 5.42.

### 3.3. Reaction of Rosamine ***4*** with Amines

(a) Protocol at 4 °C with *n*-butylamine: To an ice-bath cooled solution of rosamine **4** (9.9 mg, 23 mmol) in chloroform (500 μL), *n*-butylamine (34.3 μL, 0.35 mol, 15 equiv.) was added dropwise and the mixture was stirred at 0 °C for 2 h. Afterward, the reactional mixture was placed in the refrigerator for 22 h. After that time, the reaction mixture was washed with diethyl ether to remove the *n*-butylamine excess and purified by preparative TLC using a chloroform/methanol mixture (9:1). Amino-pyronin **5** was obtained as a yellow solid (24% yield, 2.2 mg), whereas initial rosamine **4** (22%, 2.4 mg) was recovered. A similar procedure was performed using methanol as the solvent, with an extended reaction time to six days. Amino-pyronin **5** was obtained as a yellow solid (53% yield, 4.8 mg), whereas no rosamine **4** was recovered. A similar experiment using rosamine **3** in chloroform at 4 °C was performed but, in this case, a complex mixture of products was obtained. 

(b) Protocol under MW with *n*-butylamine: A solution of rosamine **4** (9.9 mg, 23 mmol) in NMP (*N*-methylpyrrolidine, 0.1 mL) was placed in a 10 mL reaction vial, to which *n*-butylamine (34.0 μL, 0.35 mol, 15 equiv.) was added. The vial was then sealed and placed in the cavity of a CEM microwave reactor. The reaction was irradiated at 200 °C (1 min ramp to 200 °C and 35 min hold at 200 °C, using 150 W maximum power). The reaction was controlled by TLC at 5, 20, and 35 min. The resulting mixture was purified by preparative TLC using a mixture of chloroform/methanol (8:2) and vacuum dried, affording amino-pyronin **5** (10% yield, 0.9 mg), whereas rosamine **4** (7% yield, 0.7 mg) was recovered.

Amino-pyronin **5**: (a) Yield 53% (4.8 mg) and (b) Yield 10% (0.9 mg) ^1^H NMR (600 MHz, MeOD-d_4_) δ: 1.05 (3H, t, *J* 7.4 Hz, NHCH_2_CH_2_CH_2_CH_3_), 1.29 (12H, t, *J* 6.8 Hz, 4×CH_2_CH_3_), 1.55 (2H, q, *J* 7.4 Hz, NHCH_2_CH_2_CH_2_CH_3_), 1.93 (2H, t, *J* 7.4 Hz, NHCH_2_CH_2_CH_2_CH_3_), 3.60 (8H, q, *J* 6.8 Hz, 4×CH_2_CH_3_), 4.02 (2H, t, *J* 7.4 Hz, NHCH_2_CH_2_CH_2_CH_3_), 6.73 (2H, s broad, H-4 and H-5), 6.98 (2H, dd, *J* 9.0 and *J* 1.8 Hz, H-2 and H-7), 8.12 (2H, d, *J* 8.4 Hz, H-1 and H-8) ppm. ^13^C NMR (150 MHz, MeOD-d_4_) δ: 11.3 (NHCH_2_CH_2_CH_2_CH_3_), 12.6 (CH_2_CH_3_), 19.7 and 31.2 and 47.2–48.2 (CH_2_, where one of the signals is under the signal of MeOD-d_4_), 44.5 (CH_2_CH_3_), 67.7, 81.7, 96.2 (C-4 and C-5), 110.7 (C-2 and C-7), 128.5, 153.4, 154.6. HRMS (ESI) *m/z*: calcd. for C_25_H_36_N_3_O^+^, 394.3285; found, 394.285. 

(c) Protocol at 4 °C with putrescine: To an ice-bath solution of rosamine **4** (10.2 mg, 2.4 × 10^−2^ mol) in chloroform (500 μL), putrescine (17.8 μL, 0.18 mol, 7.5 equiv.) was added dropwise and the mixture was stirred at 0 °C for 2 h. Then, the reactional mixture was placed in the refrigerator for 22 h. After that time, the reaction mixture was washed with diethyl ether to remove the amine excess and purified by preparative TLC using a chloroform/methanol mixture (9:1), to give amino-pyronin **6a** (26% yield, 2.5 mg) as a yellow solid, and only trace amount of **6b** (dimer).

Amino-pyronin **6a**: Yield 26% (2.5 mg) ^1^H NMR (600 MHz, MeOD-d_4_) δ: 1.28 (12H, t, *J* 6.8 Hz, 4×CH_2_CH_3_), 1.83 and 2.01 (4H, 2t, *J* 7.2 Hz, NHCH_2_CH_2_CH_2_CH_2_NH_2_), 3.02 (2H, t, *J* 7.2 Hz, NHCH_2_CH_2_CH_2_CH_2_NH_2_), 3.59 (8H, q, *J* 7.2 Hz, 4×CH_2_CH_3_), 4.08 (2H, t, *J* 7.2 Hz, NHCH_2_CH_2_CH_2_CH_2_NH_2_), 6.73 (2H, s broad, H-4 and H-5), 6.97 (2H, dd, *J* 9.3 and *J* 2.4 Hz, H-2 and H-7), 8.13 (2H, d, *J* 9.3 Hz, H-1 and H-8) ppm. MS (MALDI) *m/z*: calcd for C_25_H_37_N_4_O^+^, 409.296; found, 409.409. 

(d) Protocol at 4 °C with cadaverine: To an ice-bath cooled solution of rosamine **4** (10.2 mg, 2.4 × 10^−2^ mol) in chloroform (500 μL), cadaverine (20.8 μL, 0.18 mol, 7.5 equiv.) was added dropwise and the mixture was stirred at 0 °C for 2 h. Afterward, the reactional mixture was placed in the refrigerator for 22 h. After that time, the reaction mixture was washed with diethyl ether to remove the amine excess and purified by preparative TLC using a chloroform/methanol mixture (9:1) to give amino-pyronin **7a** (15% yield, 1.5 mg) and **7b** (0.6 mg, 3%).

Amino-pyronin **7a**: Yield 15% (1.5 mg) ^1^H NMR (600 MHz, MeOD-d_4_) δ: 1.29 (12H, t, *J* 6.8 Hz, 4×CH_2_CH_3_), 1.60, 1.78 and 2.01 (6H, 3t, *J* 7.3 Hz, NHCH_2_CH_2_CH_2_CH_2_CH_2_NH_2_), 2.99 (2H, t, *J* 7.3 Hz, NHCH_2_CH_2_CH_2_CH_2_CH_2_NH_2_), 3.61 (8H, q, *J* 6.8 Hz, 4×CH_2_CH_3_), 4.06 (2H, t, *J* 7.3 Hz, NHCH_2_(CH_2_)_4_NH_2_), 6.74 (2H, s broad, H-4 and H-5), 6.99 (2H, dd, *J* 9.0 and *J* 2.1 Hz, H-2 and H-7), 8.15 (2H, d, *J* 9.0 Hz, H-1 and H-8) ppm. MS (MALDI) *m/z*: calcd. for C_26_H_39_N_4_O^+^, 423.312; found, 423.450. 

Amino-pyronin **7b**: Yield 3% (0.6 mg) ^1^H NMR (400 MHz, MeOD-d_4_) δ: 1.26 (24H, t, *J* 7.2 Hz, 8×CH_2_CH_3_), 1.71 (2H, t, *J* 7.3 Hz, NHCH_2_CH_2_CH_2_CH_2_CH_2_NH), 2.04 (4H, t, *J* 7.3 Hz, NHCH_2_CH_2_CH_2_CH_2_CH_2_NH), 3.57 (16H, q, *J* 7.2 Hz, 8×CH_2_CH_3_), 4.05 (4H, t, *J* 7.3 Hz, NHCH_2_(CH_2_)_3_CH_2_NH_2_), 6.66 (4H, d, *J* 2.5 Hz, H-4 and H-5), 6.89 (4H, dd, *J* 9.6 and *J* 2.5 Hz, H-2 and H-7), 8.07 (4H, d, *J* 9.6 Hz, H-1 and H-8) ppm. MS (MALDI) *m/z*: calcd. for C_47_H_63_N_6_O_2_^+^, 743.501; found, 743.969. 

### 3.4. Sensing Study of Rosamine ***4*** with Different Biogenic Amines 

(a) Preliminary studies in solution. Nine samples were prepared from 500 μL of rosamine **4** dissolved in acetonitrile with a concentration of 10^−5^ M. Then, 2, 4, and up to 8 equiv. of 5 μL solutions of biogenic amines (1-histamine, 2-tyramine, 3-cadaverine, 4-putrescine, 5-phenylethylamine, 6-spermidine, 7-spermine, 8-*n*-butylamine, and 9-rosamine **4**) dissolved in water were added. The changes were observed under visible and UV light and registered.

(b) UV-Vis and fluorescence studies in solution. A stock solution of rosamine **4** in DMSO was diluted to 35 μM in acetonitrile. Then 0.1, 0.5, 1.0, 2.0, 4.0, and 8.0 equiv. of amine solutions (*n*-butylamine, *t*-butylamine, cadaverine, and putrescine) in acetonitrile were added. UV–Vis and fluorescence spectra were recorded in the appropriate concentration and wavelength ranges between 225 and 800 nm (absorbance) and 561–800 nm (emission; *λ*_exc_ = 551 nm, 5 nm slit width for excitation and emission and 650 V) at 25 °C.

(c) Studies in gas phase. The study was carried out using 2.5 mL of rosamine **4** dissolved in acetonitrile and with a concentration of 5 μM. To dispose the amines in the gas phase, a small amount of each commercial biogenic amine (cadaverine, spermidine, spermine, phenylethylamine, histamine, tyramine, tryptamine, and putrescine) was placed in 10 mL vials, closed with a septum, and heated on a heating plate, as shown in Figure S32, at different temperatures depending on the boiling point of each amine. The vapor produced was transferred with a syringe and bubbled into a cuvette containing 2.5 mL of **4**. Afterward, the measurement was carried out in the spectrophotometer at 533 nm. The same procedure was carried out using nitrogen, air, and water vapor to check any putative effects on probe **4**.

## 4. Conclusions

Two catechol-derived rosamine dyes were synthesized through a simple and straightforward strategy involving the microwave-assisted synthesis of rosamines **1** and **2**, with subsequent debenzylation using boron trichloride to give derivatives **3** and **4**. Significant changes in the UV–Vis spectra were observed for the catechol derived rosamine **4** toward biogenic amines (BAs), especially for putrescine, where two new bands appeared at 512 and 649 nm and a quench was observed at 551 nm. The highest fluorescence intensity quenching was observed for the addition of 8 equiv. of putrescine (55%).

Furthermore, we found that methanolic solutions of rosamine **4** and *n*-butylamine showed an expressive color change over time, which was attributed to the formation of the 9-aminopyronin **5**. Different parameters including solvent, temperature, and reaction time influence the outcome of this transformation; the best yield was obtained in methanol at 4 °C for ca. 144 h, affording **5** in 53% yield. Other 9-aminopyronins **6a**, **6b**, **7a**, and **7b** were obtained from methanolic solutions of **4** with putrescine and cadaverine. All 9-aminopyronins exhibited exceptional spectroscopic properties with blue shifts in absorption and emission spectra as well as a high increase in the fluorescence quantum yield, comparatively to the pristine rosamine **4**. 

The present results corroborate the high reactivity of the catechol derivative **4** toward amines, and hint that this rosamine can be considered in the future as a biogenic amine sensor in solution (acetonitrile) and in the gas phase.

## Figures and Tables

**Table 1:** Photophysical properties (*λ*_max abs_, *λ*_max em_, *ε*, Stokes shift, and *Φ*_F_) of rosamines **1**–**4** and **RhB** in chloroform, acetonitrile, and methanol at 25 °C.

Dye	R	Solvent	λ_max (abs)_ (nm)	*ε* × 10^4^ (M^−1^·cm^−1^)	λ_max (em)_ (nm)	Stokes Shift (nm) (cm^−1^)	*Φ*_F_
**1**	2,3-OBn	CHCl_3_	560	7.01	576	16 (496)	0.60
		CH_3_CN	560	8.66	582	22 (675)	0.23
		CH_3_OH	558	5.68	580	22 (680)	0.30
**2**	3,4-OBn	CHCl_3_	556	7.44	569	13 (411)	0.58
		CH_3_CN	555	6.18	576	21 (657)	0.20
		CH_3_OH	554	6.36	573	19 (689)	0.25
**3**	2,3-OH	CHCl_3_	546	10.5	566	20 (647)	0.04
		CH_3_CN	557	4.50	580	23 (712)	0.12
		CH_3_OH	550	9.38	570	20 (638)	0.02
**4**	3,4-OH	CHCl_3_	548	5.06	570	22 (704)	0.04
		CH_3_CN	552	5.10	573	21 (664)	0.12
		CH_3_OH	553	4.94	573	20 (631)	0.03
**RhB**	2-COOH	CHCl_3_	554	11.2	571	17 (537)	0.54
		CH_3_CN	555	8.20	578	23 (717)	0.22
		CH_3_OH	547	11.5	569	22 (707)	0.33

**Table 2:** Photophysical properties (*λ*_max abs_, *λ*_max em_, *ε*, Stokes shift, and *Φ*_F_) of aminopyronin **5**, **6a**, **7a**, **7b** in chloroform, acetonitrile, and methanol at 25 °C.

Dye.	Solvent	*λ*_max abs_ (nm)	*ε* × 10^4^ (M^−1^·cm^−1^)	*λ*_max em_ (nm)	Stokes Shift (nm) (cm^−1^)	*Φ*_F_
**5**	CHCl_3_	435	5.31	507	72 (3265)	0.57
	CH_3_CN	436	4.35	523	87 (3815)	0.10
	CH_3_OH	438	5.43	525	87 (3783)	0.19
**6a**	CHCl_3_	430	7.44	507	77 (3532)	0.36
	CH_3_CN	440	6.46	525	85 (3680)	0.17
	CH_3_OH	439	6.75	522	83 (3622)	0.24
**7a**	CHCl_3_	434	9.57	512	78 (3510)	0.58
	CH_3_CN	438	8.01	524	86 (3747)	0.11
	CH_3_OH	438	8.91	522	84 (3674)	0.20
**7b**	CHCl_3_	435	10.8	508	73 (3303)	0.53
	CH_3_CN	438	9.21	526	88 (3820)	0.16
	CH_3_OH	440	10.6	524	84 (3643)	0.21

## Data Availability

The data presented in this study are contained within the article and are also available in the Supplementary Materials.

## Supplementary Materials

The following are available online: MS, ^1^H, ^13^C, and 2D NMR spectra of the new compounds; additional UV–Vis and fluorescence measurements including the fluorescence intensity of **4** with pH variation and detection of biogenic amines in solution and in the gas phase.

## Sample Availability

Samples of compounds **1–7** are available from the authors.
