# Supplementary material for: Synthesis of Catechol Derived Rosamine Dyes and Their Reactivity toward Biogenic Amines
Source: Molecules. 2021 Aug 22;26(16):5082. doi: 10.3390/molecules26165082 (PMC8401866; doi:10.3390/molecules26165082)
Supplement: Supplementary file 1 [file molecules-26-05082-s001.zip › molecules-1335012-supplementary.pdf]

Supplementary Materials: Synthesis of Catechol Derived Rosamine Dyes and Their Reactivity toward Biogenic Amines

Filipe Monteiro-Silva [1,†], Carla Queirós [2,†], Andreia Leite [2], María Teresa Rodríguez [3], María José Rojo [3], Tomás Torroba [3], Rui C. Martins [1], Ana M. G. Silva [2,*] and Maria Rangel [4]

1 Centre for Applied Photonics, INESC TEC, Faculdade de Ciências da Universidade do Porto, Rua do Campo Alegre, 4169-007 Porto, Portugal; filipe.m.silva@inesctec.pt (F.M.-S.);
rui.c.martins@inesctec.pt (R.C.M.)

2 LAQV/REQUIMTE, Departamento de Química e Bioquímica, Faculdade de Ciências da Universidade do Porto, Rua do Campo Alegre, 4169-007 Porto, Portugal; carla.que iros@fc.up.pt (C.Q.);
acleite@fc.up.pt (A.L.)

3 Departamento de Química, Facultad de Ciencias, Universidad de Burgos, 09001 Burgos, España; mtrod@ubu.es (M.T.R); mjrocam@ubu.es (M.J.R.); ttorroba@ubu.es (T.T.)

4 LAQV/REQUIMTE, Instituto de Ciências Biomédicas de Abel Salazar, Rua Jorge de Viterbo Ferreira 228, 4050-313 Porto, Portugal; mrangel@icbas.up.pt

* Correspondence: ana.silva@fc.up.pt

† These authors contributed equally to this work.

* Author to whom correspondence should be addressed: Tel: (+351)220402585 Fax: (+351)220402659; email: ana.silva@fc.up.pt

Contents

NMR spectrum of 2,3-dibenzyloxybenzaldehyde and 3,4-dibenzyloxybenzaldehyde 2
NMR spectra of rosamine 1 3
NMR spectra of rosamine 2 6
NMR spectra of rosamine 3 8
NMR spectra of rosamine 4 11
MS spectra of 1, 2, 3 and 4 13
The influence of pH variation in the fluorescence intensity of rosamine 4. 15
Detection study of rosamine 4 with different biogenic amines in solution (preliminary study) 16
Detection study of rosamine 4 with different amines in solution (CH_3CN) 18
Detection study of rosamine 4 with different biogenic amines in gas phase 19
NMR spectra of aminopyronin 5 20
^{1}H NMR spectrum comparison between rosamine 4 and aminopyronin 5 21
NMR spectrum of aminopyronin 6a 22
NMR spectrum of aminopyronin 7a 22
NMR spectrum of aminopyronin 7b 23
MS spectra of 5, 6a, 7a and 7b 23

NMR spectrum of 2,3-dibenzyloxybenzaldehyde and 3,4-dibenzyloxybenzaldehyde

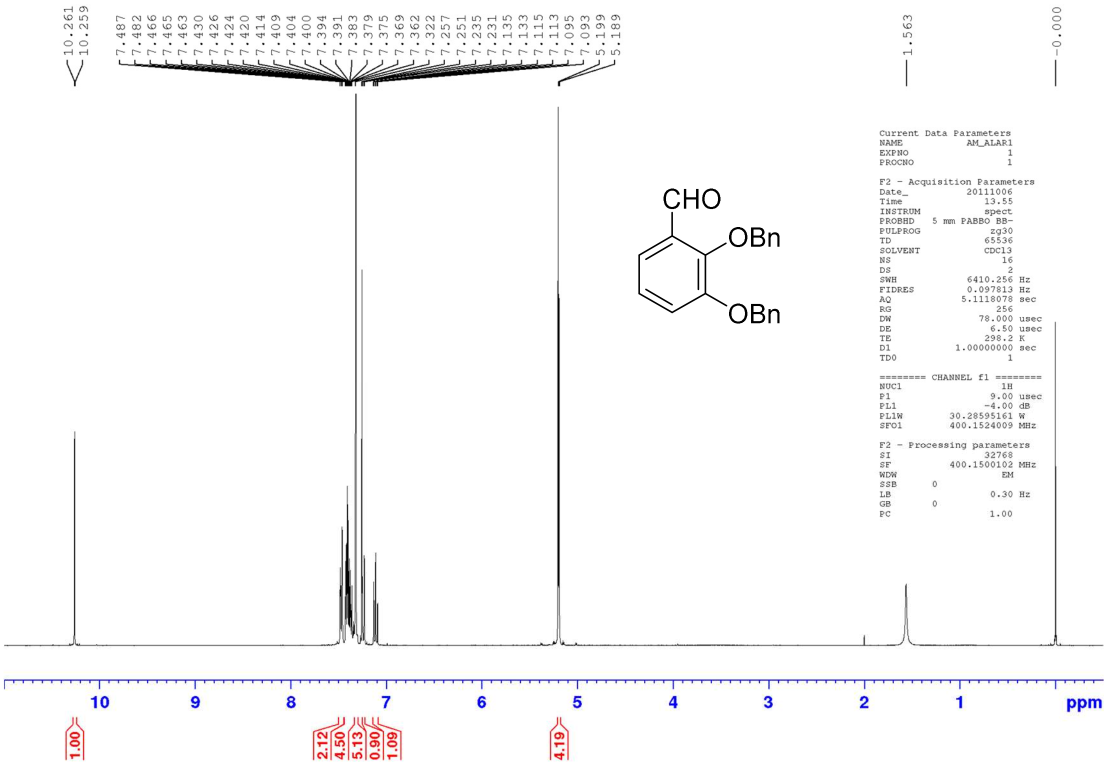

Figure S1. ^{1}H NMR spectrum (400.15 MHz, $CDCl_3$) of 2,3-dibenzyloxybenzaldehyde.

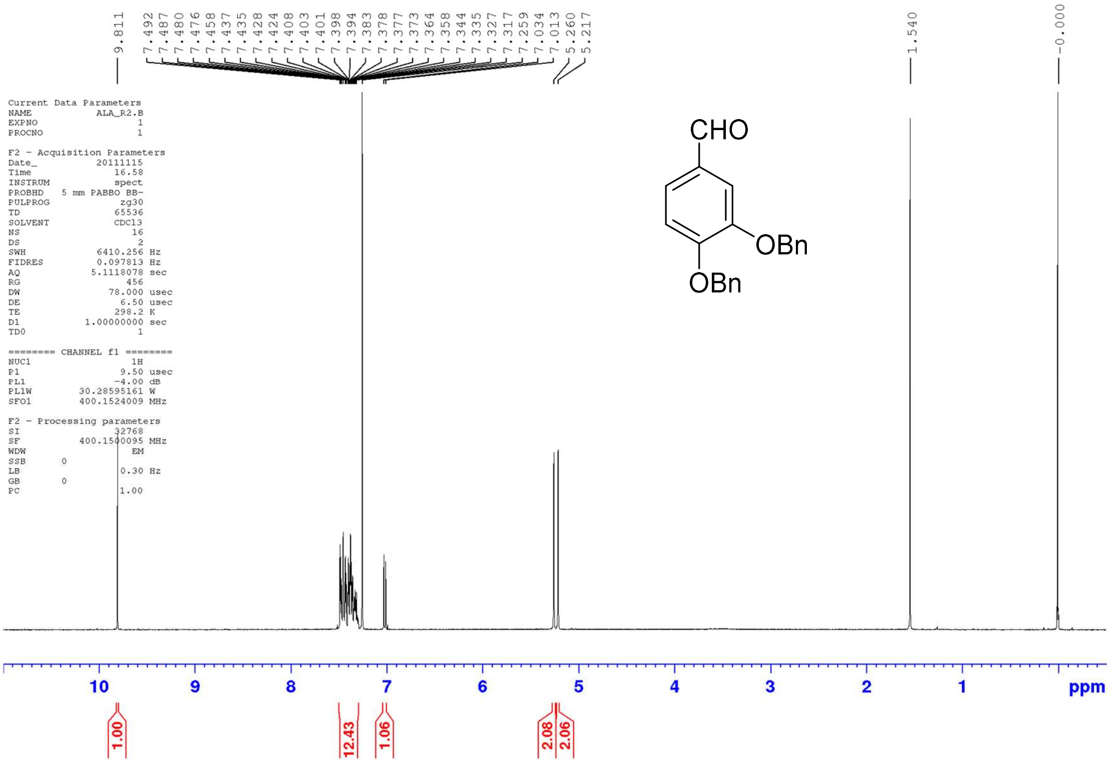

Figure S2. ^{1}H NMR spectrum (400.15 MHz, $CDCl_3$) of 3,4-dibenzyloxybenzaldehyde.

NMR spectra of rosamine 1

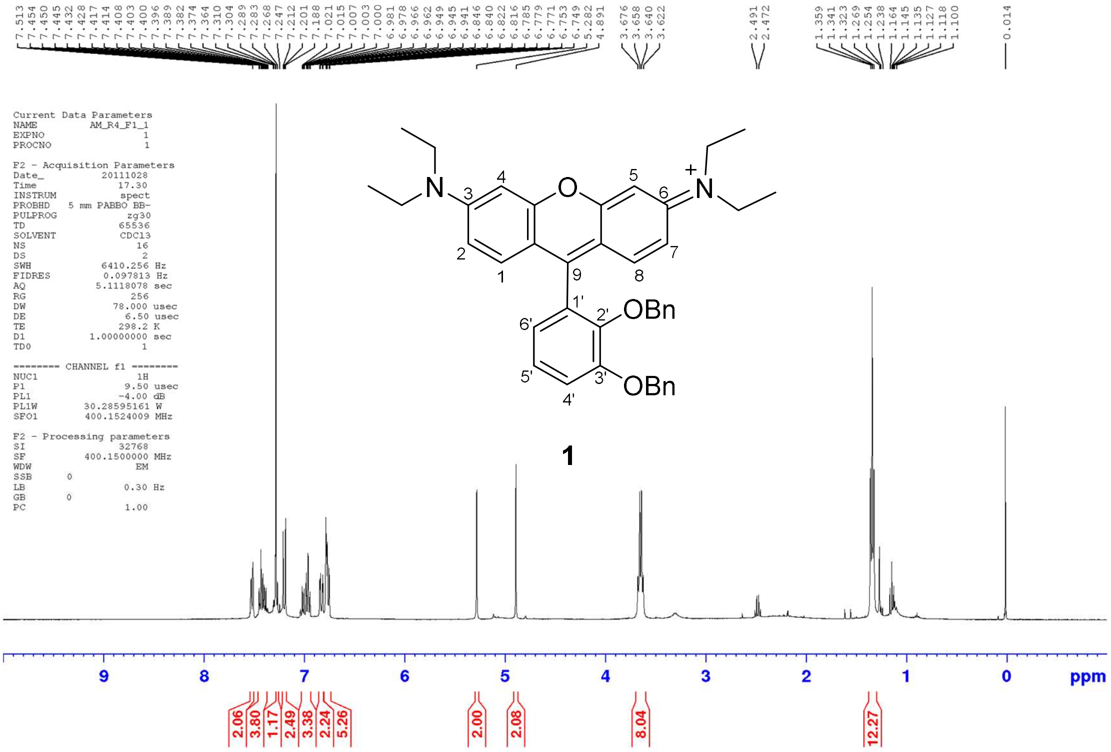

Figure S3. ^{1}H NMR spectrum (400.15 MHz, $CDCl_3$) of **1**.

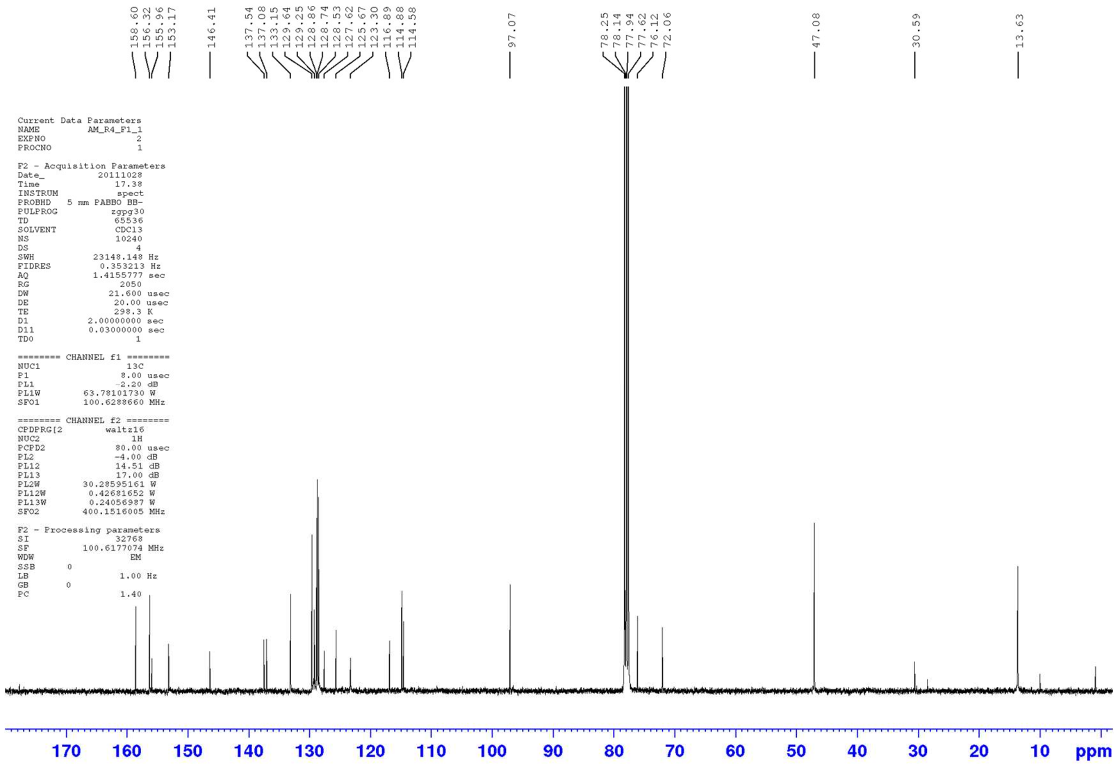

Figure S4. ^{13}C NMR spectrum (100.63 MHz, $CDCl_3$) of **1**.

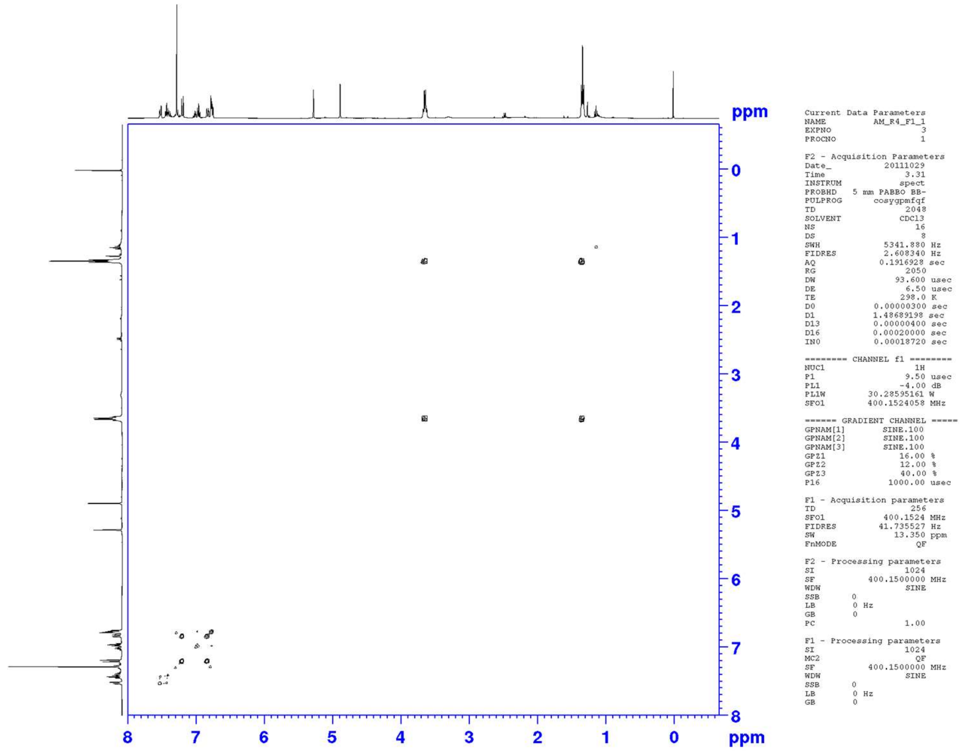

Figure S5. COSY spectrum of **1**.

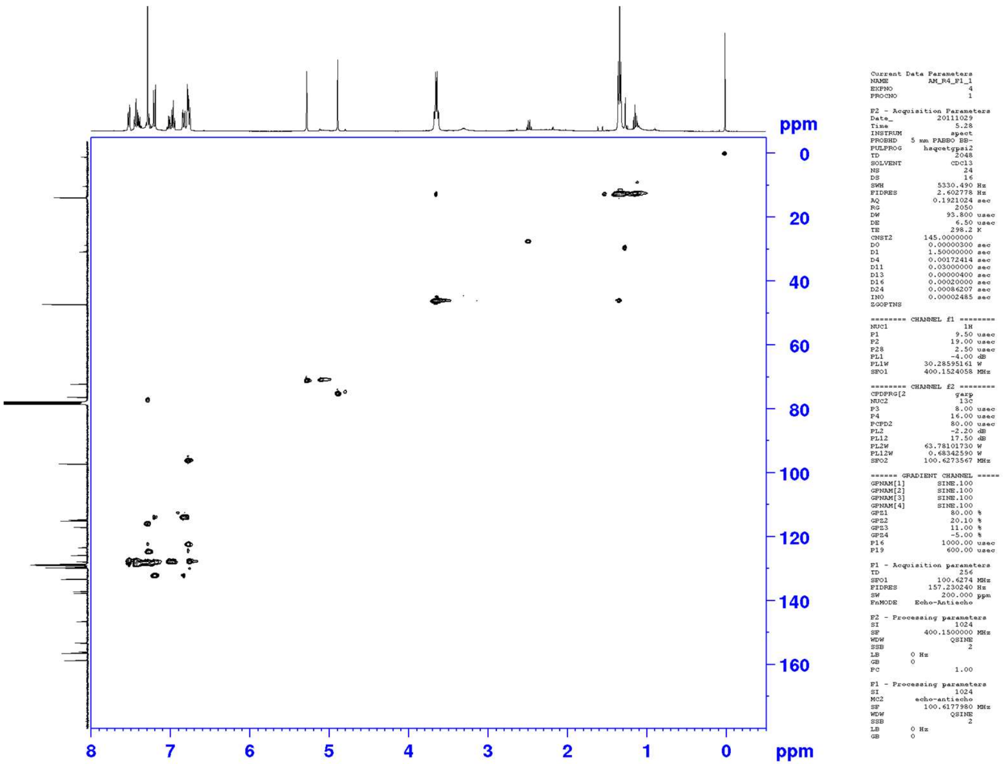

Figure S6. HSQC spectrum of **1**.

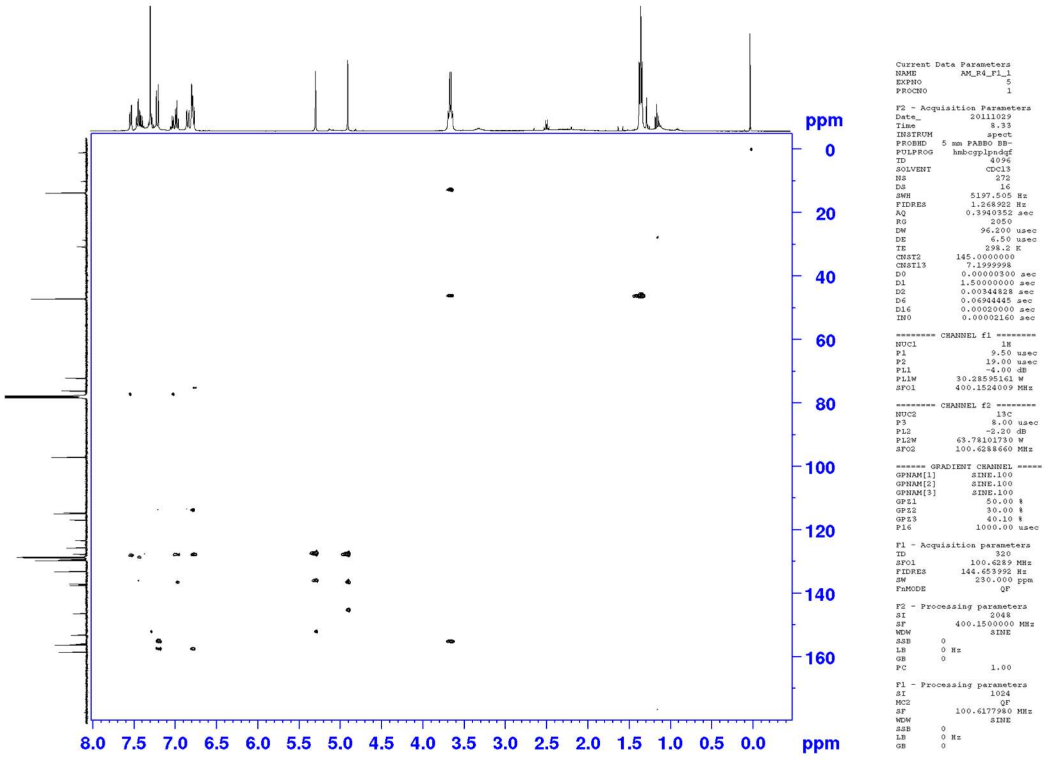

Figure S7. HMBC spectrum of **1**.

NMR spectra of rosamine 2

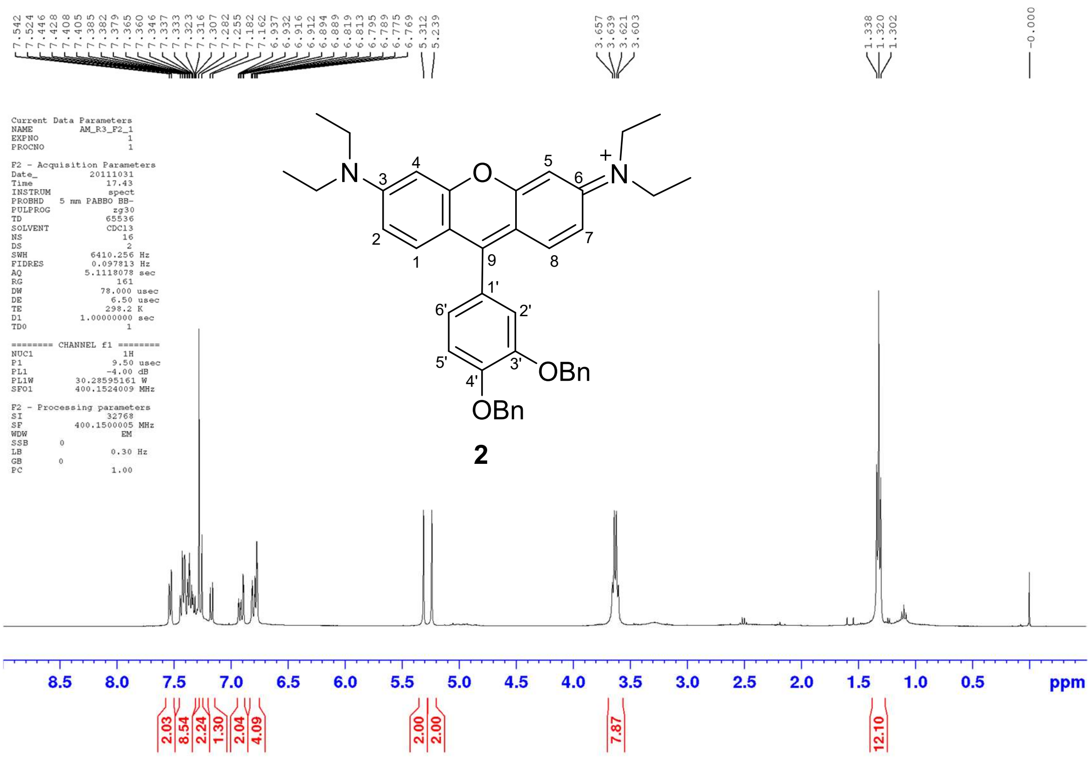

Figure S8. ^{1}H NMR spectrum (400.15 MHz, $CDCl_3$) of **2**.

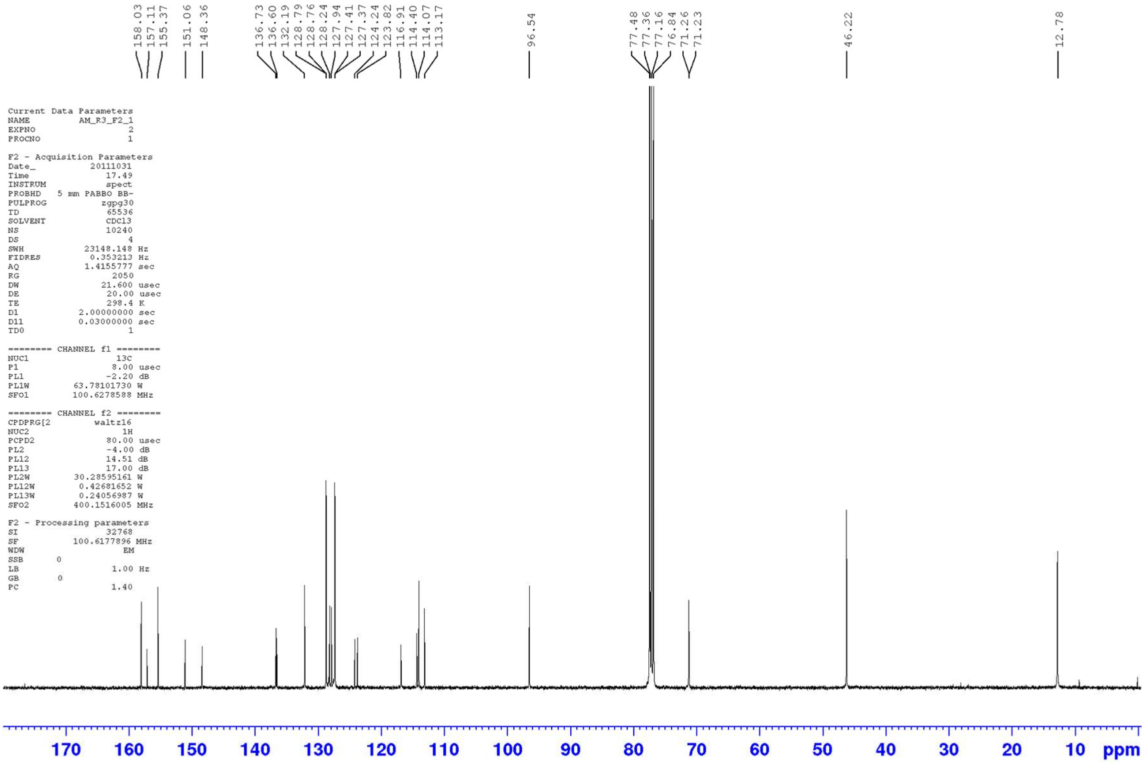

Figure S9. ^{13}C NMR spectrum (100.63 MHz, $CDCl_3$) of **2**.

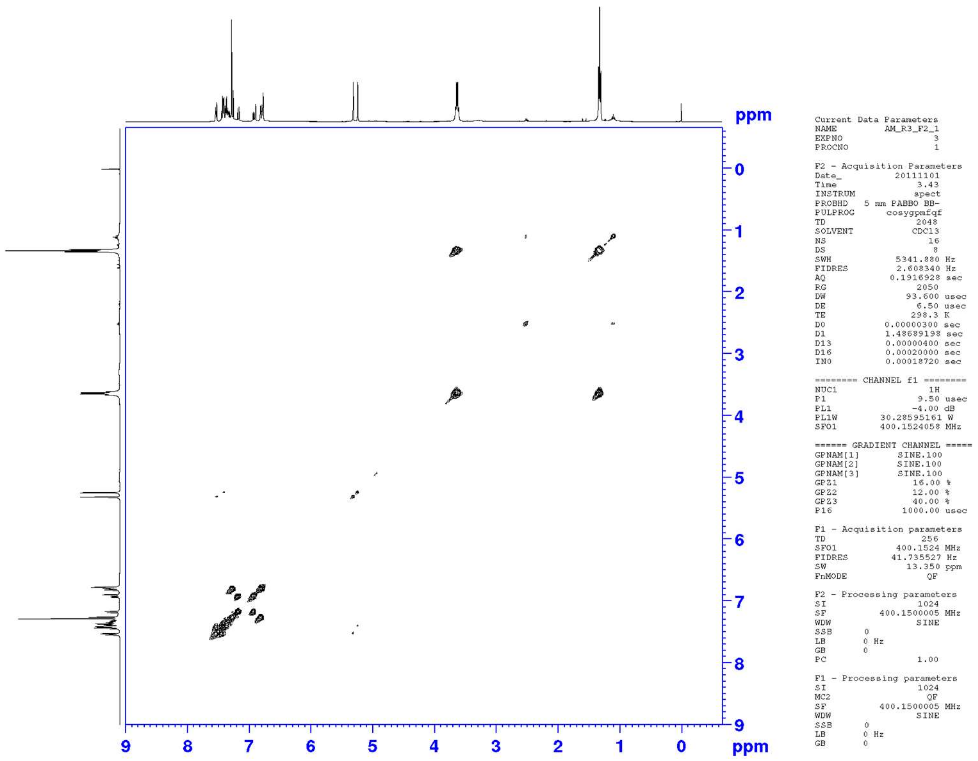

Figure S10. COSY spectrum of **2**.

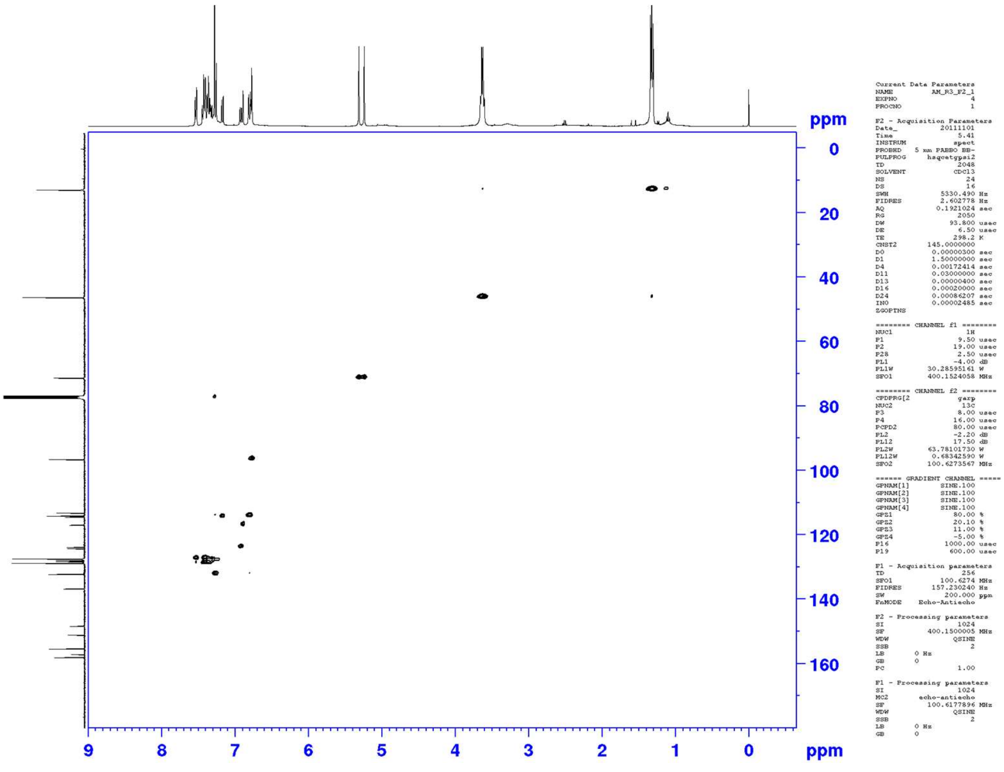

Figure S11. HSQC spectrum of **2**.

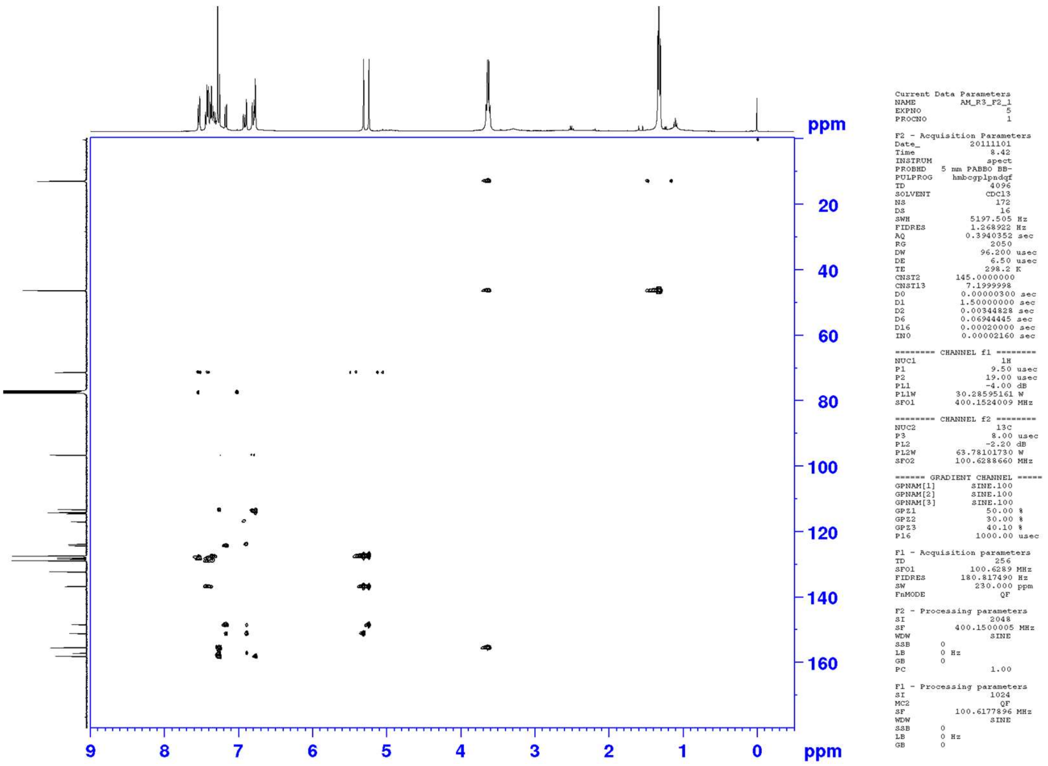

Figure S12. HMBC spectrum of **2**.

NMR spectra of rosamine 3

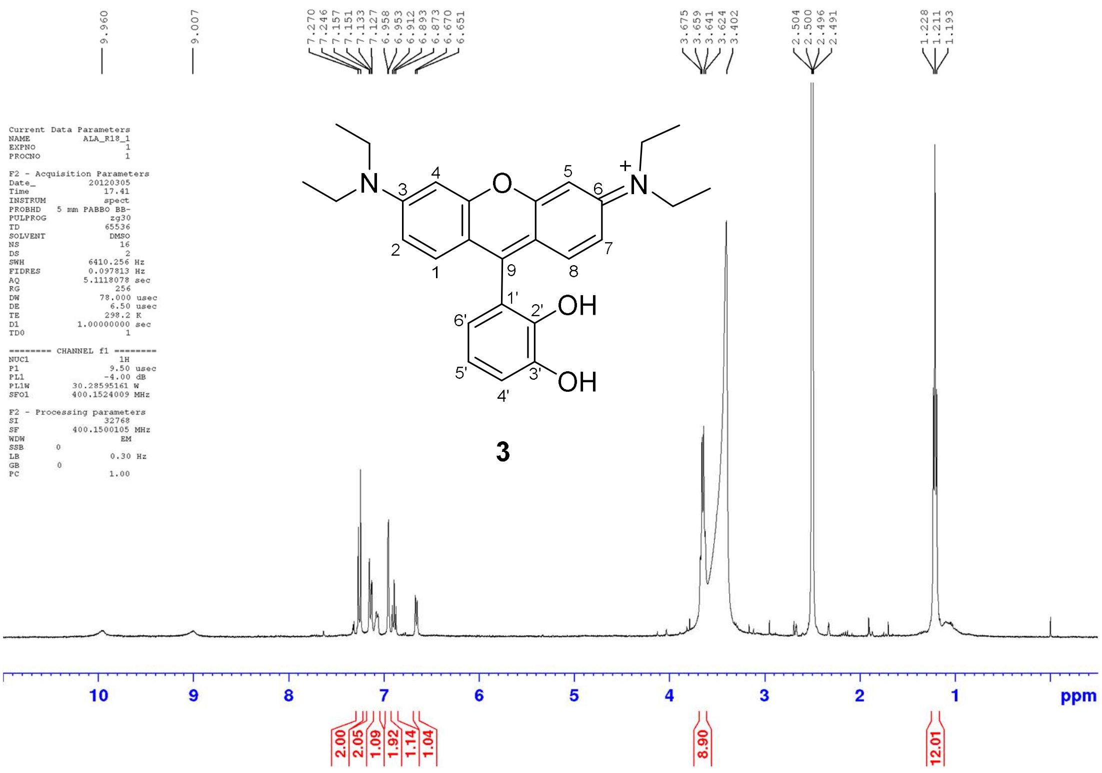

Figure S13. ^{1}H NMR spectrum (400.15 MHz, DMSO-d_6) of **3**.

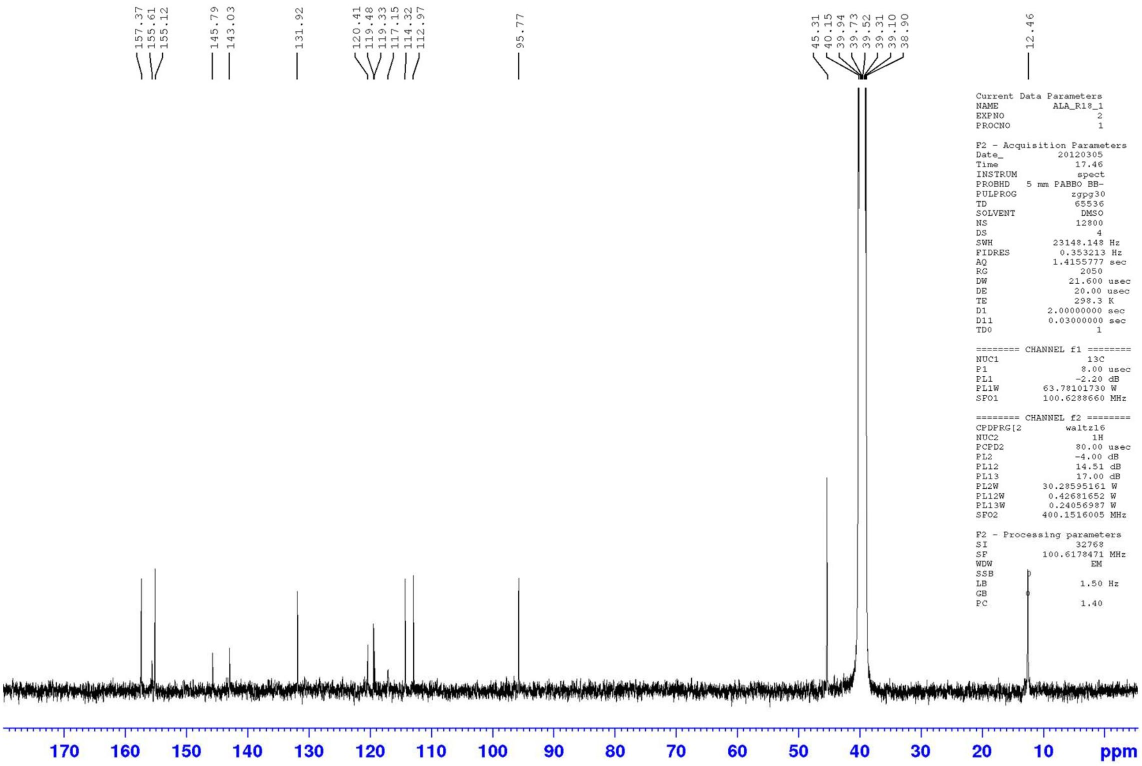

Figure S14. ^{13}C NMR spectrum (100.63 MHz, DMSO-d_6) of **3**.

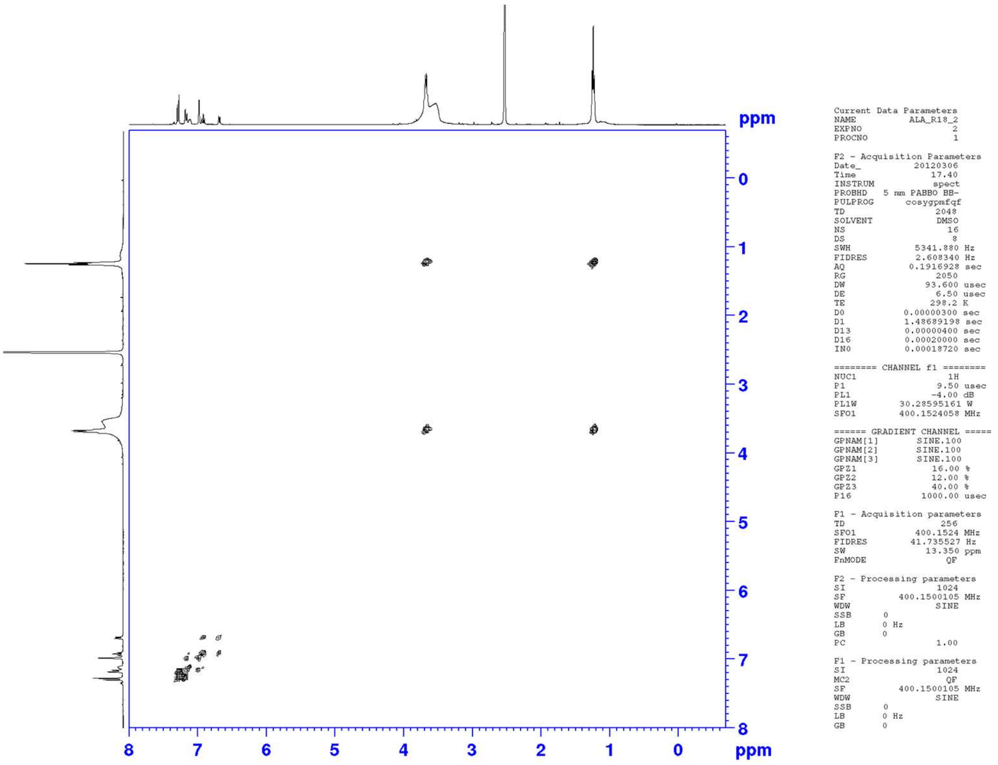

Figure S15. COSY spectrum of **3**.

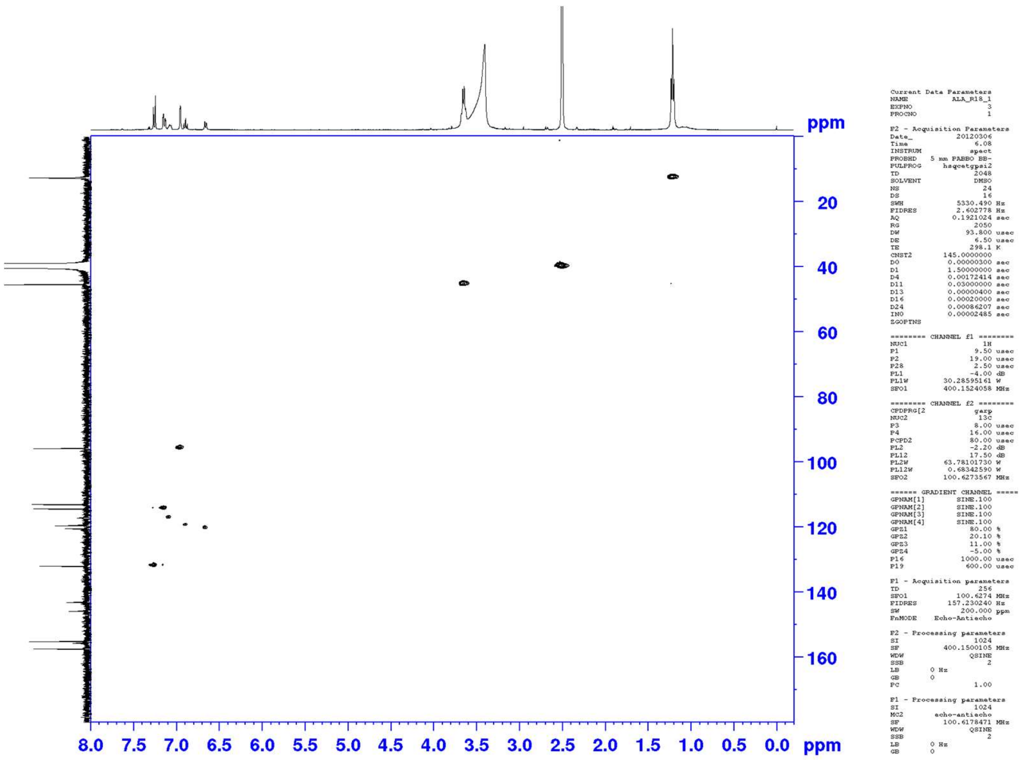

Figure S16. HSQC spectrum of **3**.

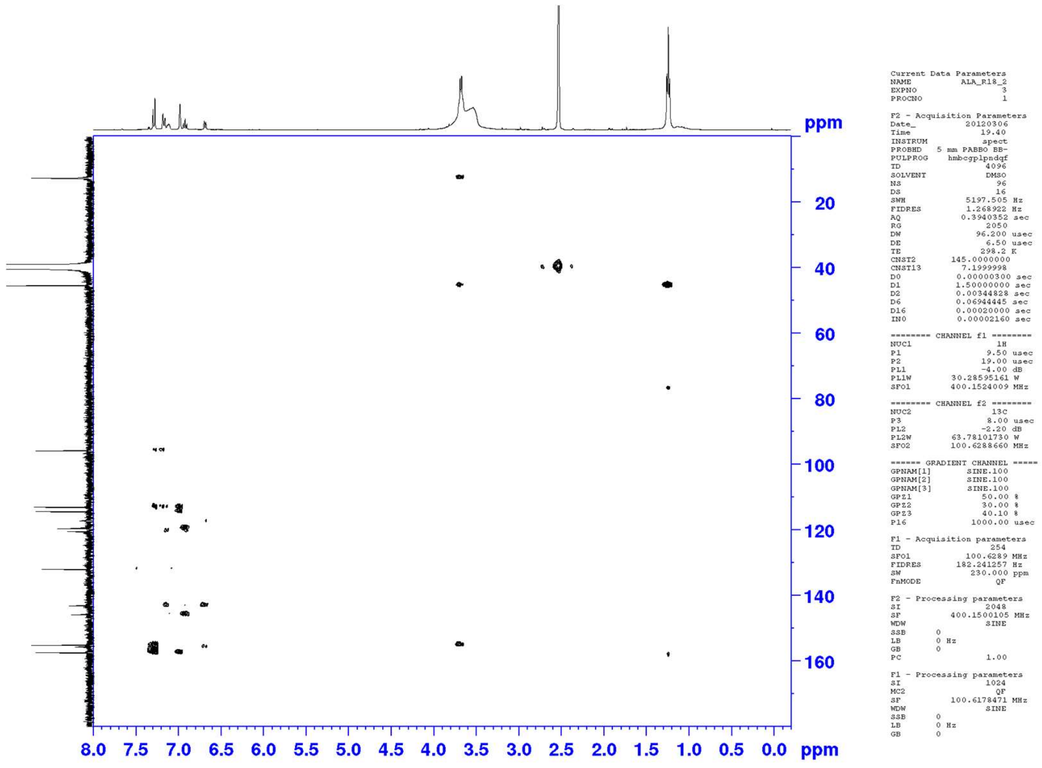

Figure S17. HMBC spectrum of **3**.

NMR spectra of rosamine 4

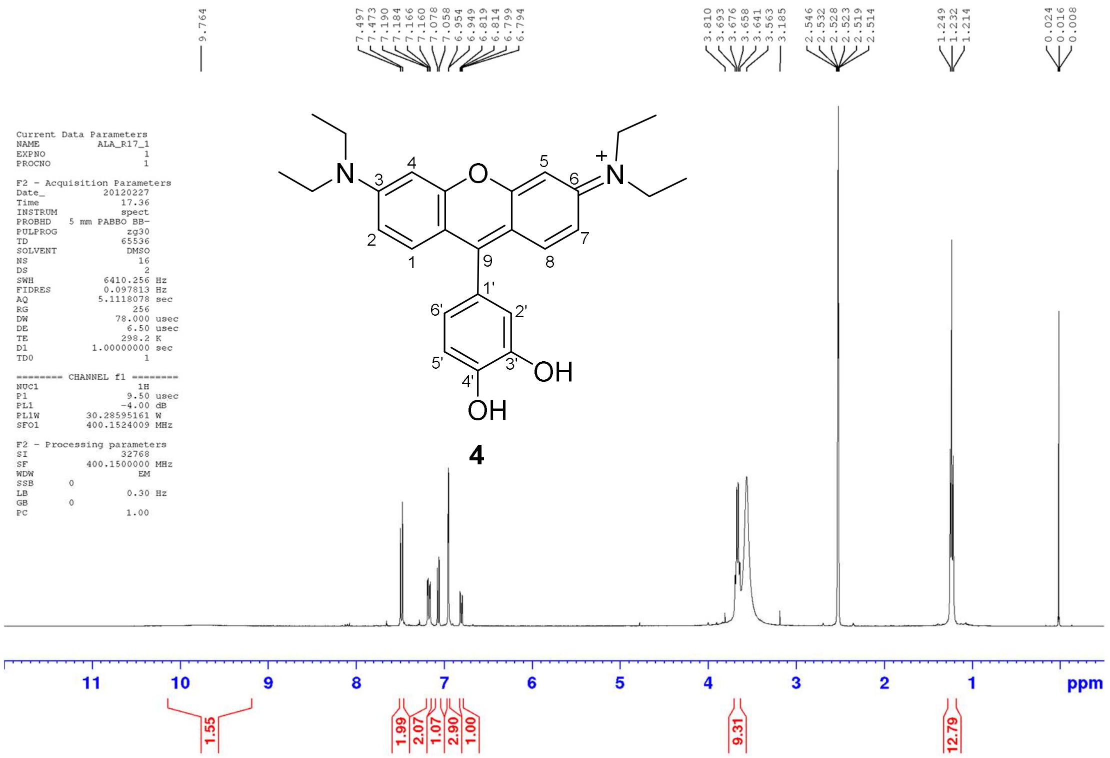

Figure S18. ^{1}H NMR spectrum (400.15 MHz, DMSO-d_6) of **4**.

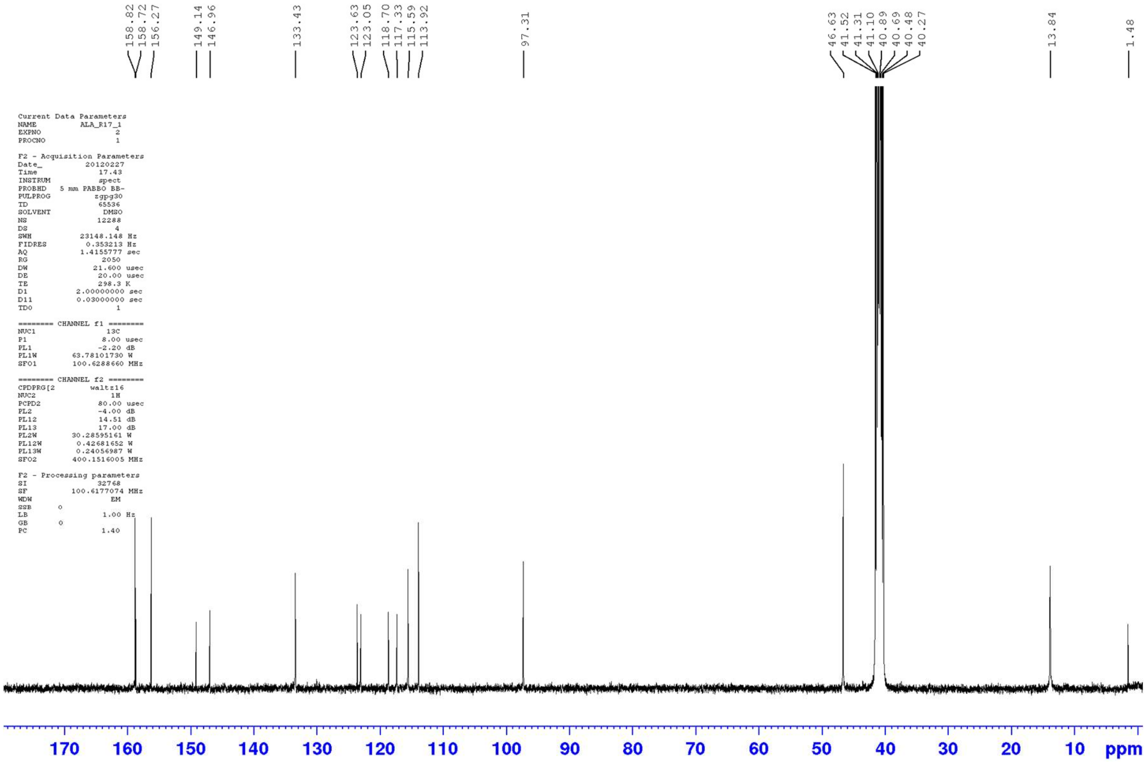

Figure S19. ^{13}C NMR spectrum (100.63 MHz, DMSO-d_6) of **4**.

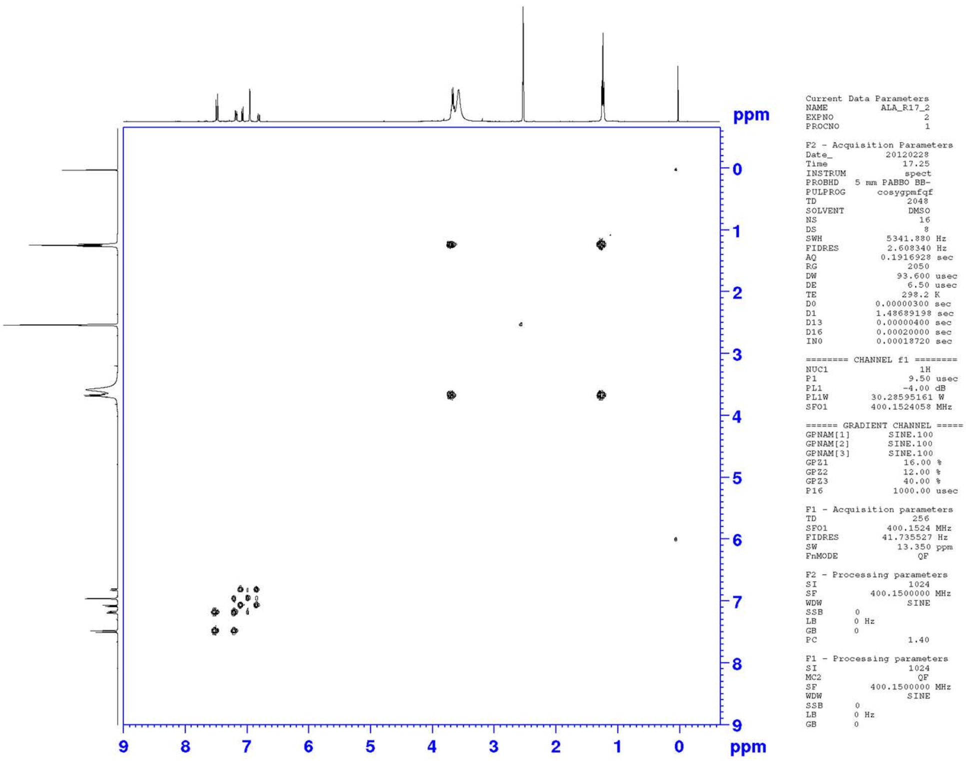

Figure S20. COSY spectrum of **4**.

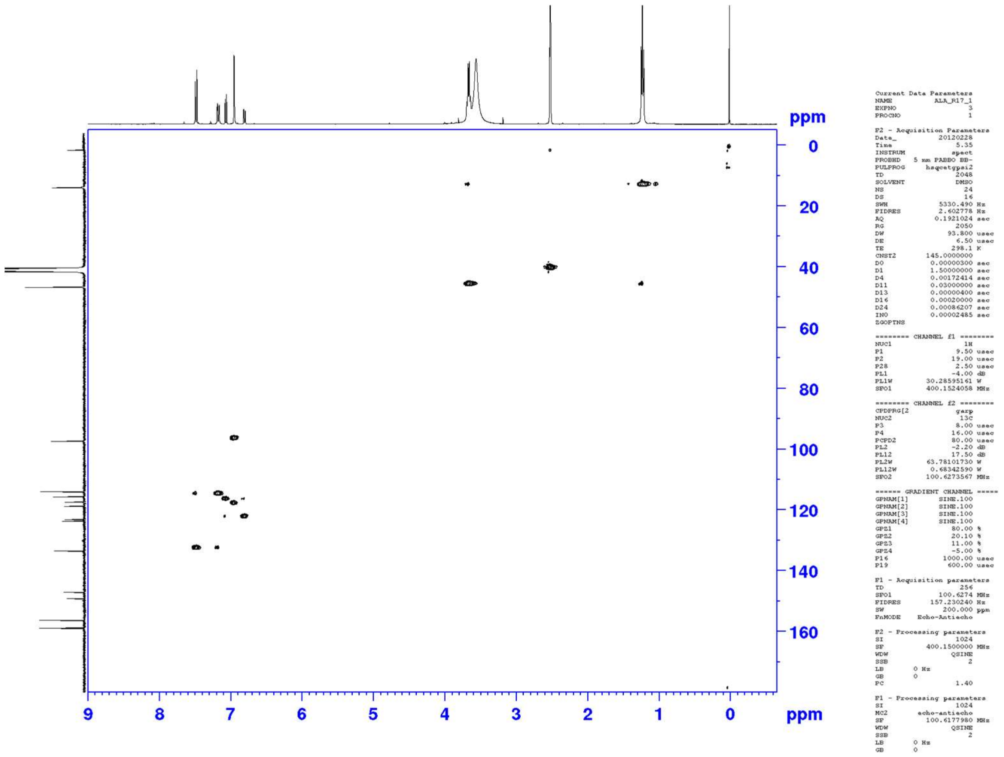

Figure S21. HSQC spectrum of **4**.

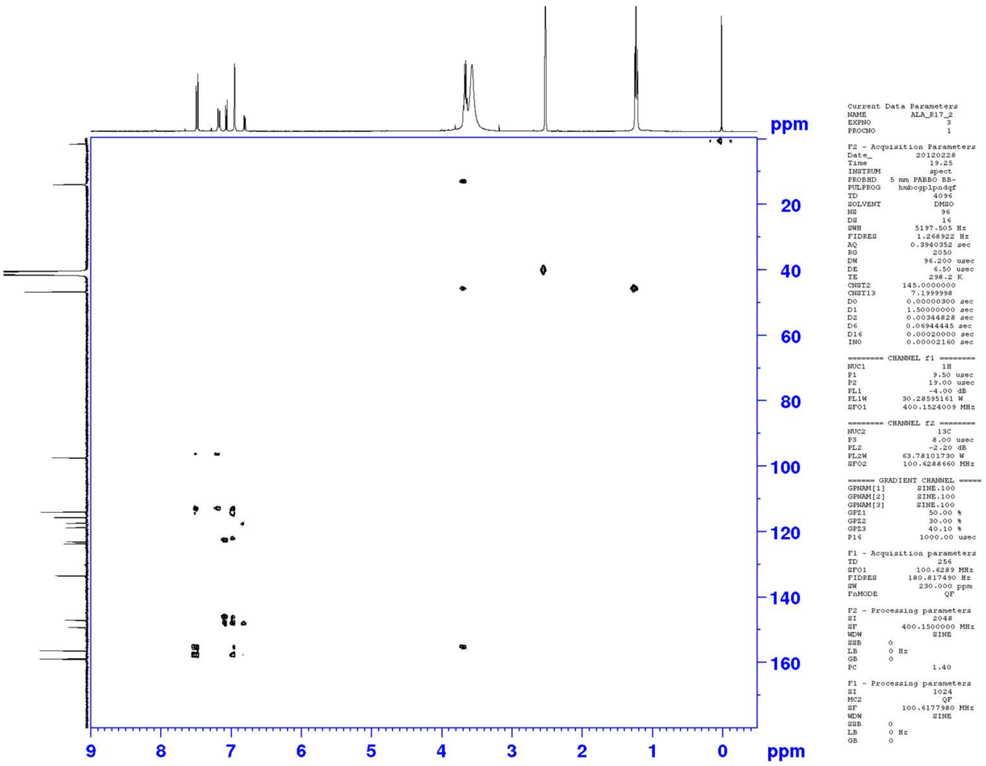

Figure S22. HMBC spectrum of **4**.

MS spectra of 1, 2, 3 and 4

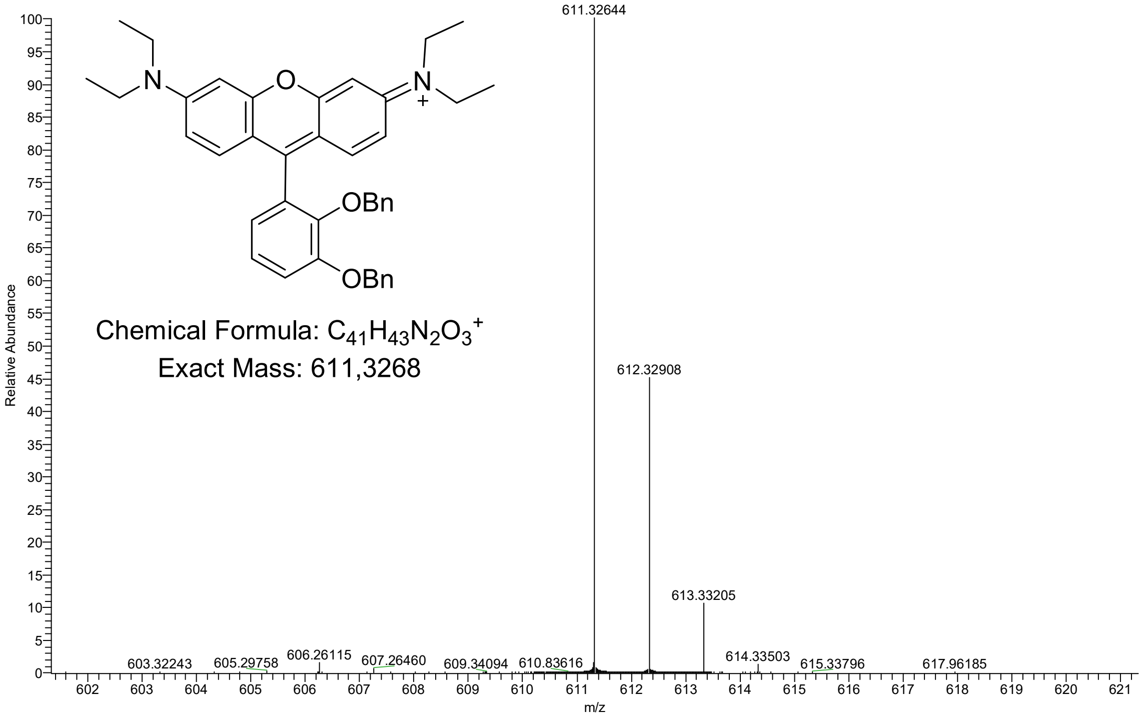

Figure S23. MS spectrum of **1**

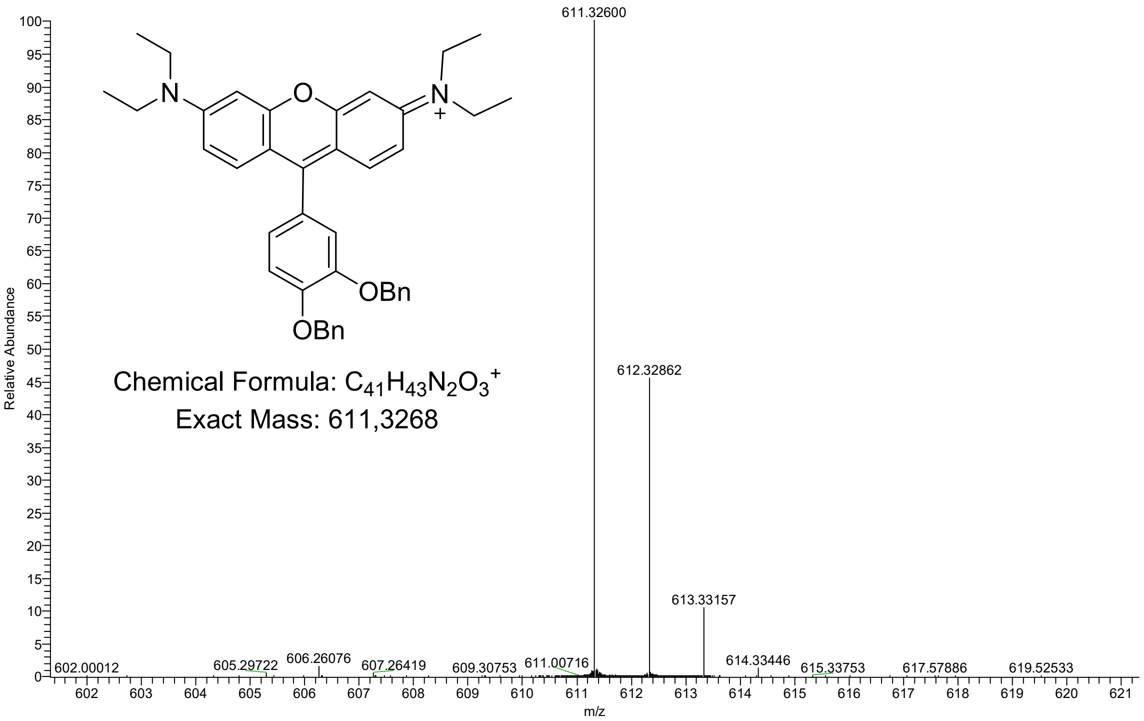

Figure S24. MS spectrum of **2**

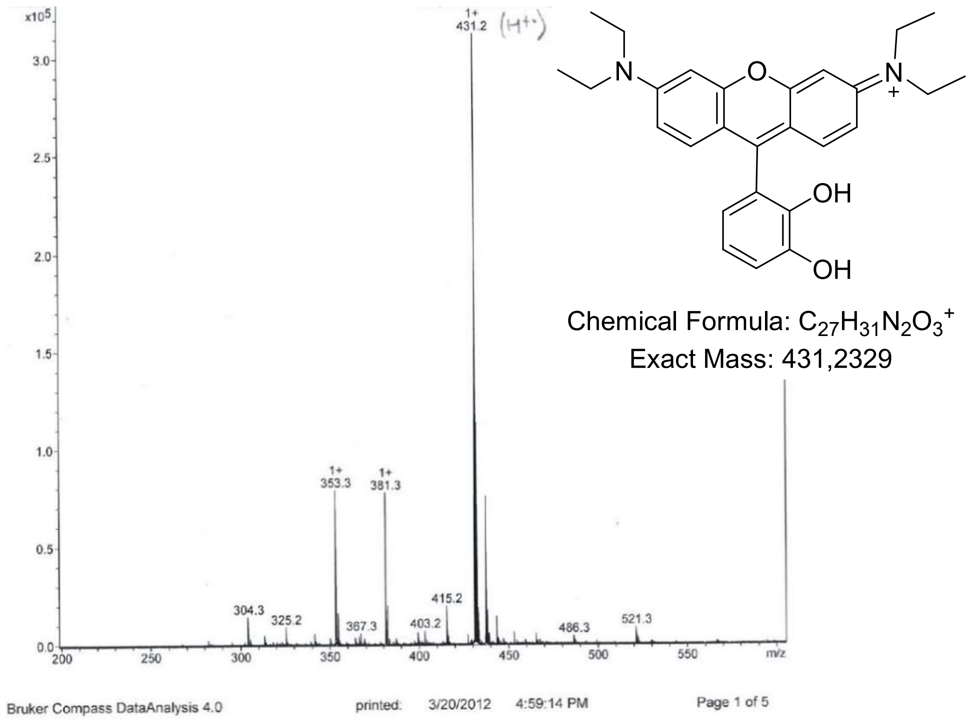

Figure S25. MS spectrum of **3**

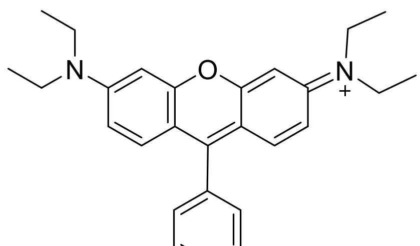

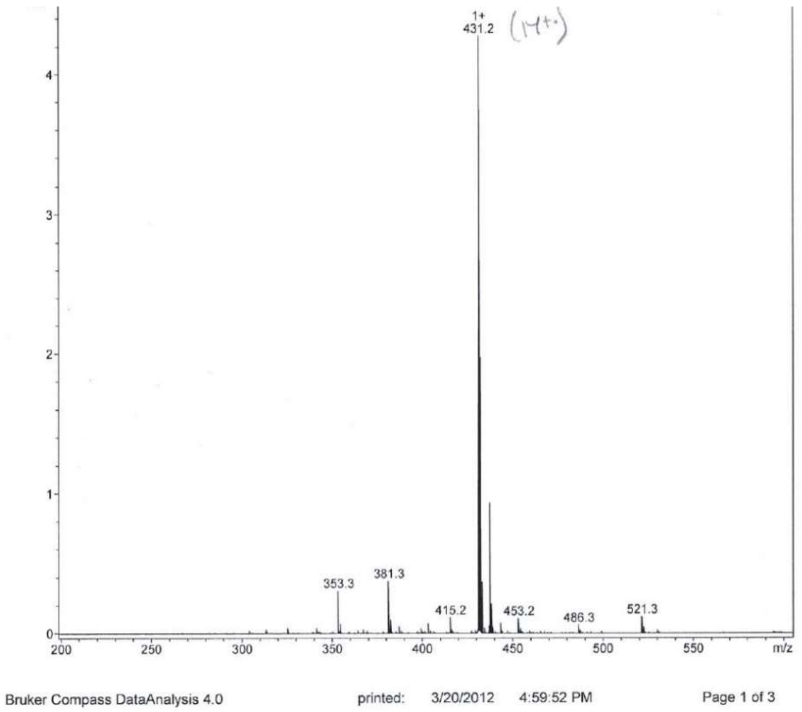

Figure S26. MS spectrum of **4**

The influence of pH variation in the fluorescence intensity of rosamine 4.

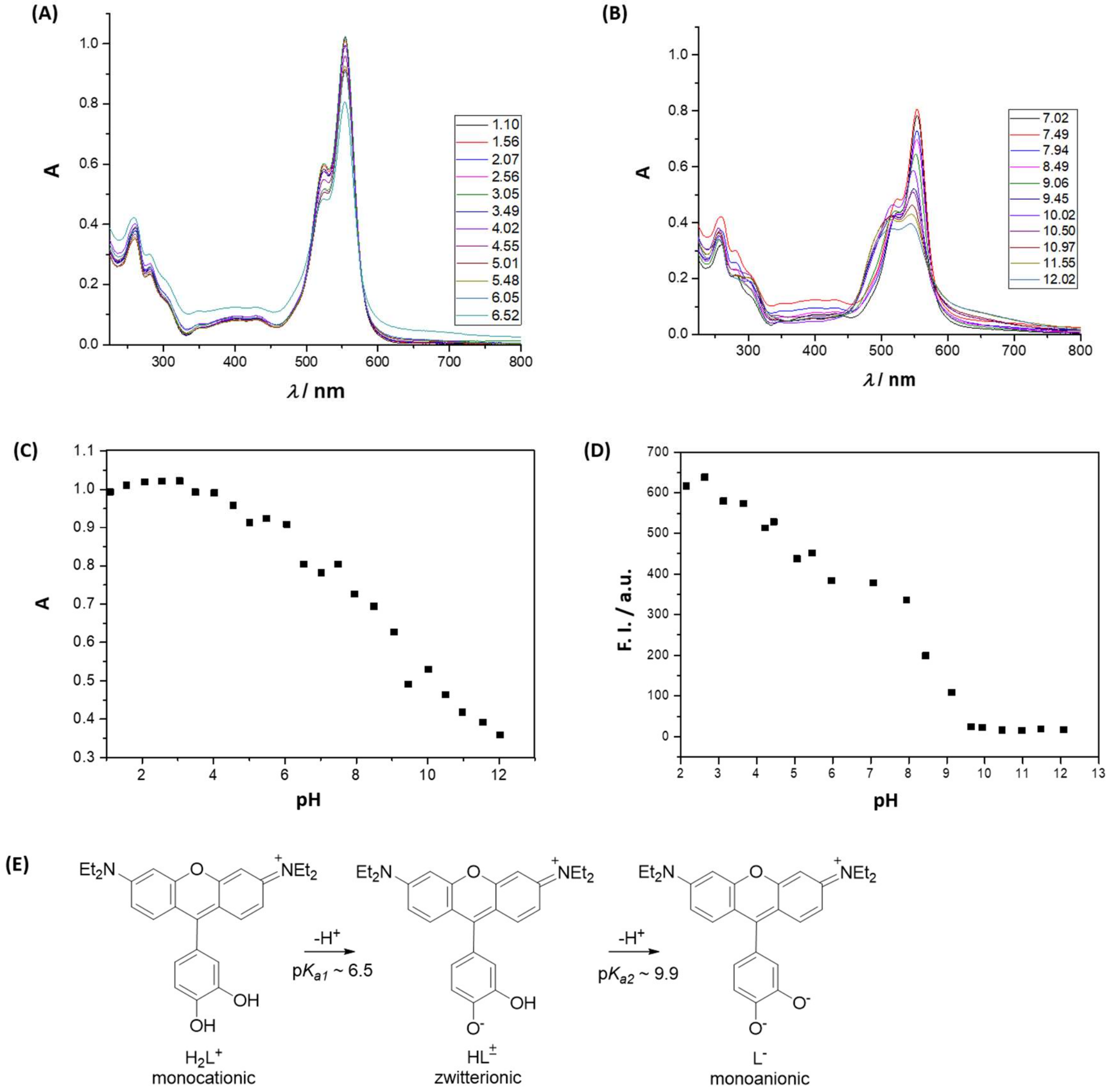

Figure S27. The influence of pH variation in the fluorescence intensity of rosamine **4**. A – Absorption spectra for pH < 7; B – Absorption spectra for pH ≥ 7); C – Graphical representation of the pH in function of the maximum in absorbance; D – Graphical representation of the pH as a function of the maximum in fluorescence intensity; E – Structures and pK_a's of rosamine **4** deprotonation.

Detection study of rosamine 4 with different biogenic amines in solution (preliminary study)

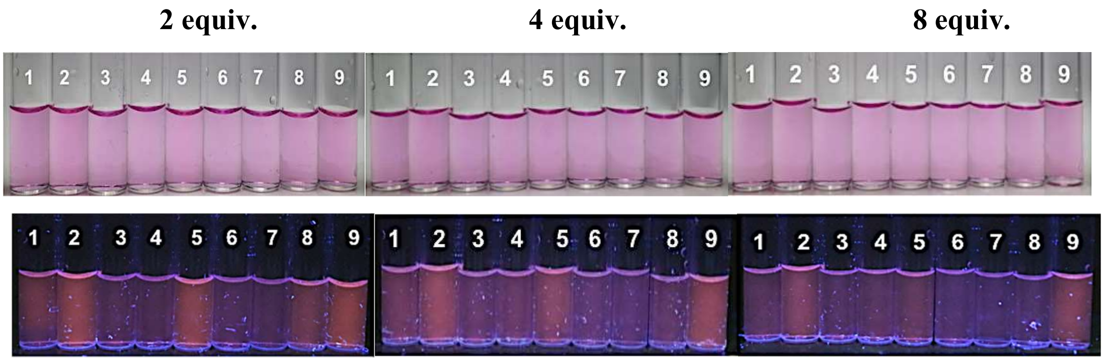

Figure S28. Samples with 2, 4 and 8 equiv. of 1-histamine, 2-tyramine, 3-cadaverine, 4-putrescine, 5-phenylethylamine, 6-spermidine, 7-spermine, 8-butylamine, 9- rosamine **4**, observed under visible (top) and UV light (bottom).

Detection study of rosamine 4 with different amines in solution (CH_3CN)

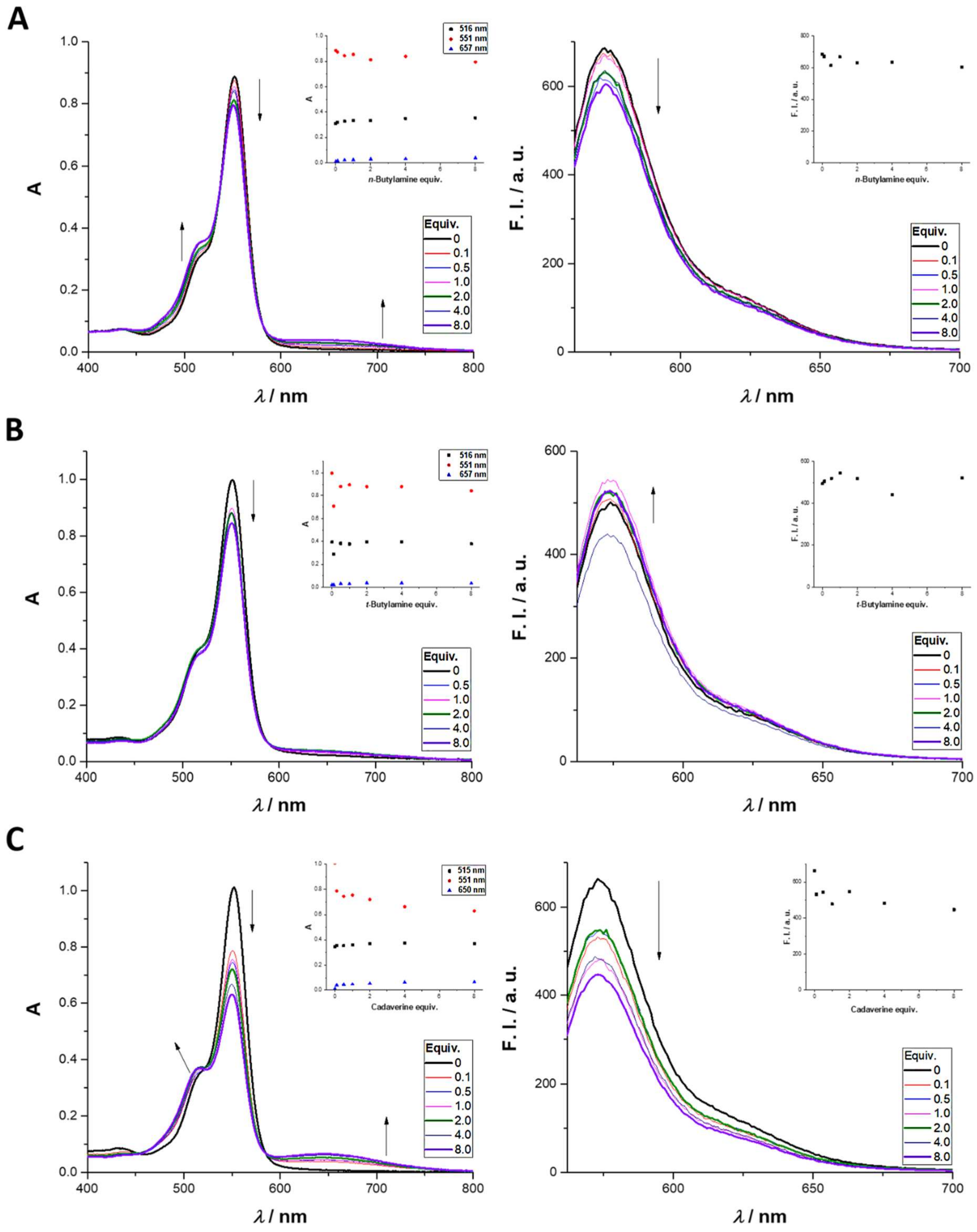

Figure S29. Absorption (left) and emission (right) spectra of **4** upon addition (0 – 8 equiv.) of *n*-butylamine (**A**), *t*-butylamine (**B**) and cadaverine (**C**) in CH_3CN (the inset graphs represent the variation in absorbance (right) and fluorescence intensity (left) for the wavelengths that present more variation in intensity. |**4**| = 35 μM and λ_{exc} = 551 nm.

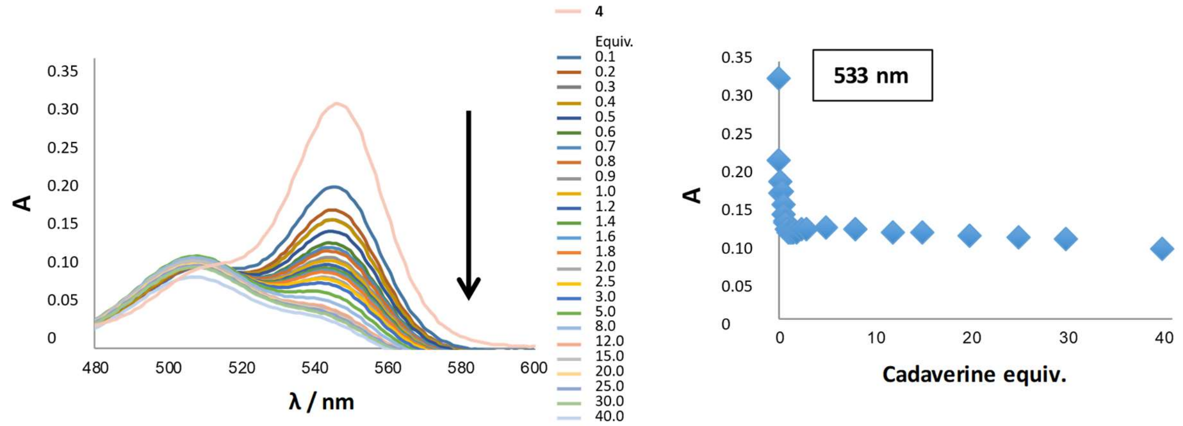

Figure S30. Absorption (left) spectrum of **4** upon addition (0.1–40 equiv.) of cadaverine in CH_3CN and graph representing the variation in absorbance (right) intensity at 533 nm |4| = 5 μM.

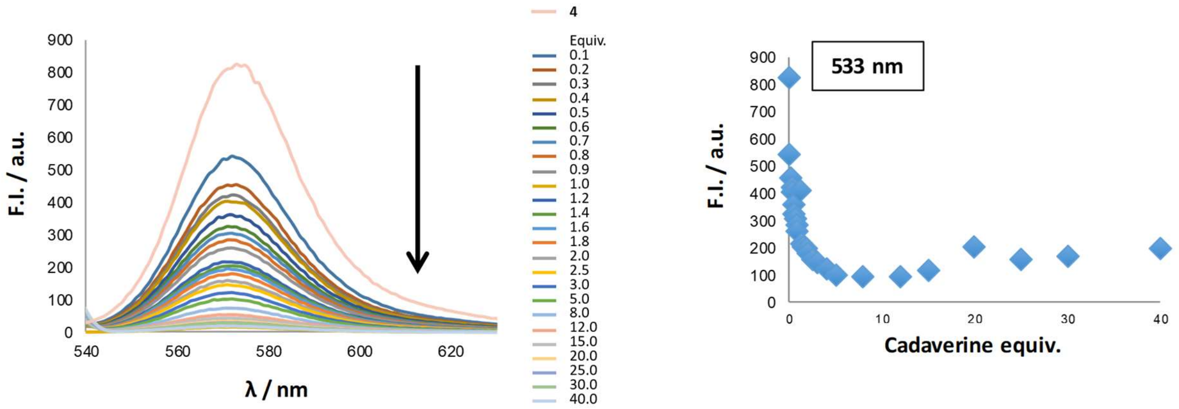

Figure S31. Emission (left) spectrum of **4** upon addition (0.1–40 equiv.) of cadaverine in CH_3CN and graph representing the variation in emission (right) intensity |4| = 5 μM and λ_{exc} = 533 nm.

Detection study of rosamine 4 with different biogenic amines in gas phase

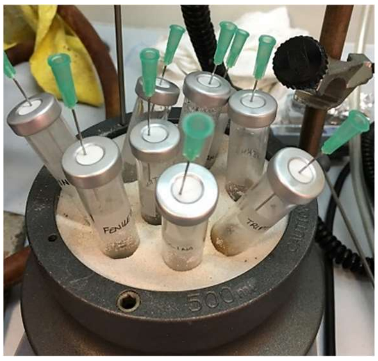

Figure S32. Commercial biogenic amine samples subjected to heat

NMR spectra of aminopyronin 5

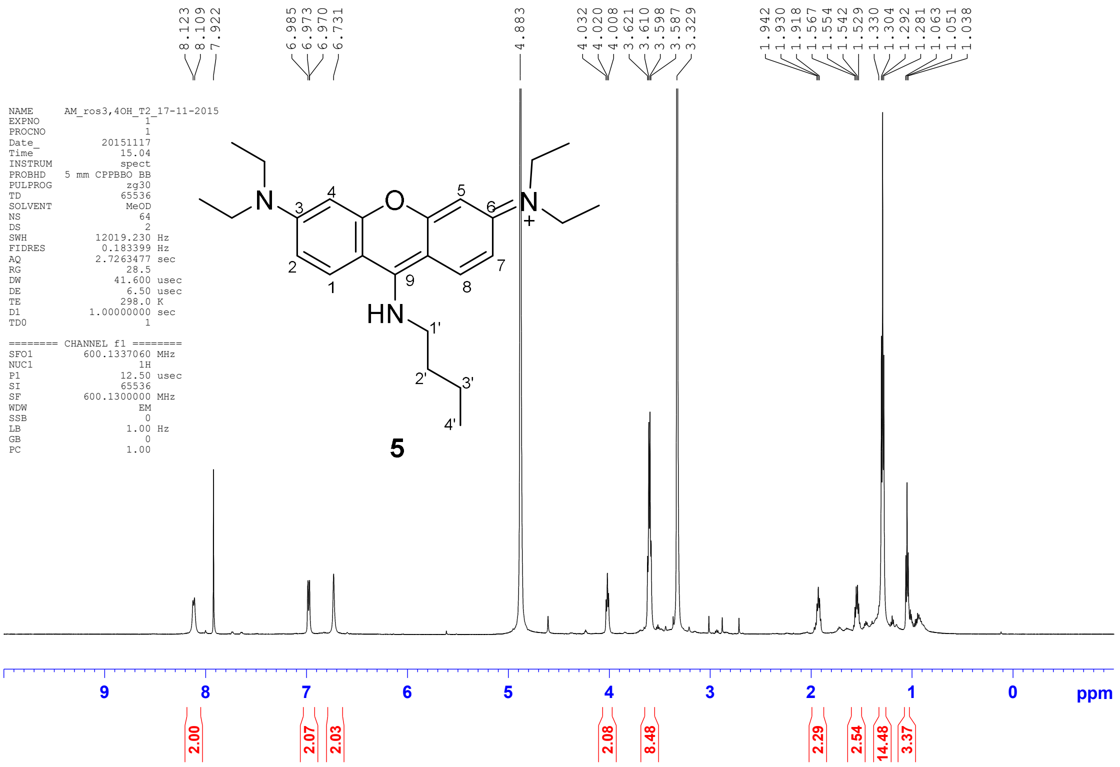

Figure S33. ^{1}H NMR spectrum (600.13 MHz, MeOD-d_4) of **5**.

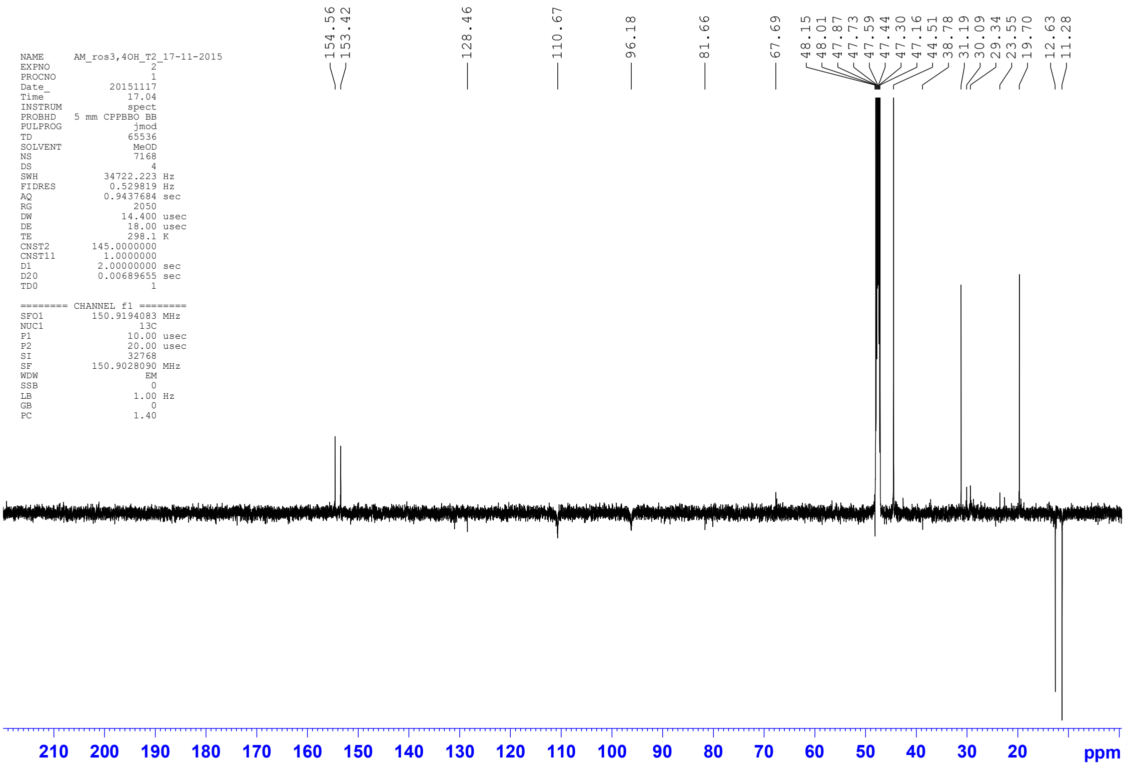

Figure S34. APT spectrum (150.90 MHz, MeOD-d_4) of **5**.

^{1}H NMR spectrum comparison between rosamine 4 and aminopyronin 5

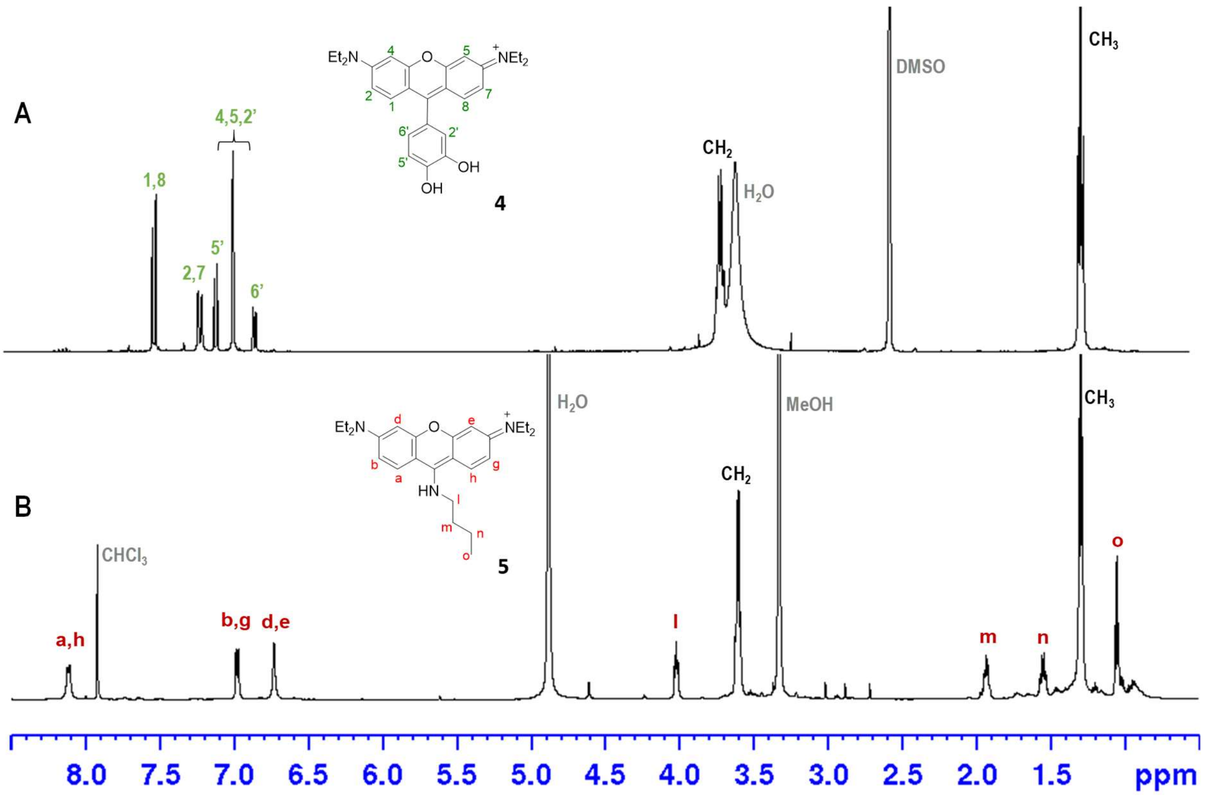

Figure S35. ^{1}H NMR spectra of **4** (A) and **5** (B).

NMR spectrum of aminopyronin 6a

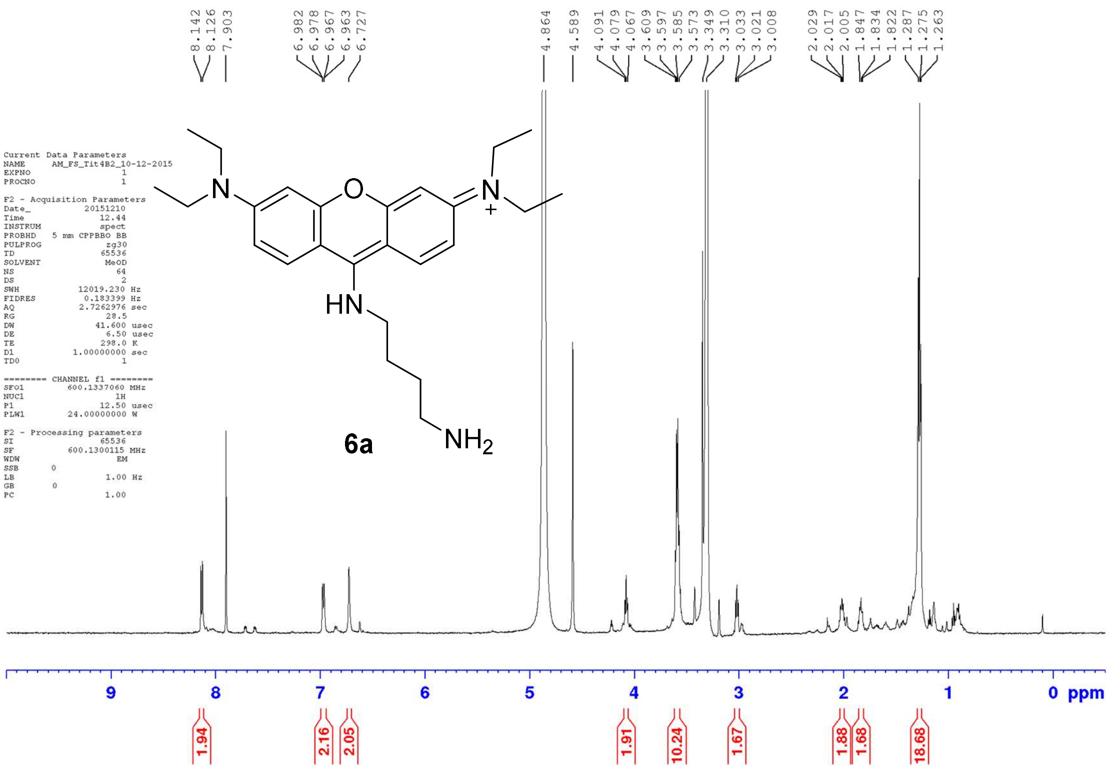

Figure S36. ^{1}H NMR spectrum (600.13 MHz, MeOD-d$_4$) of **6a**.

NMR spectrum of aminopyronin 7a

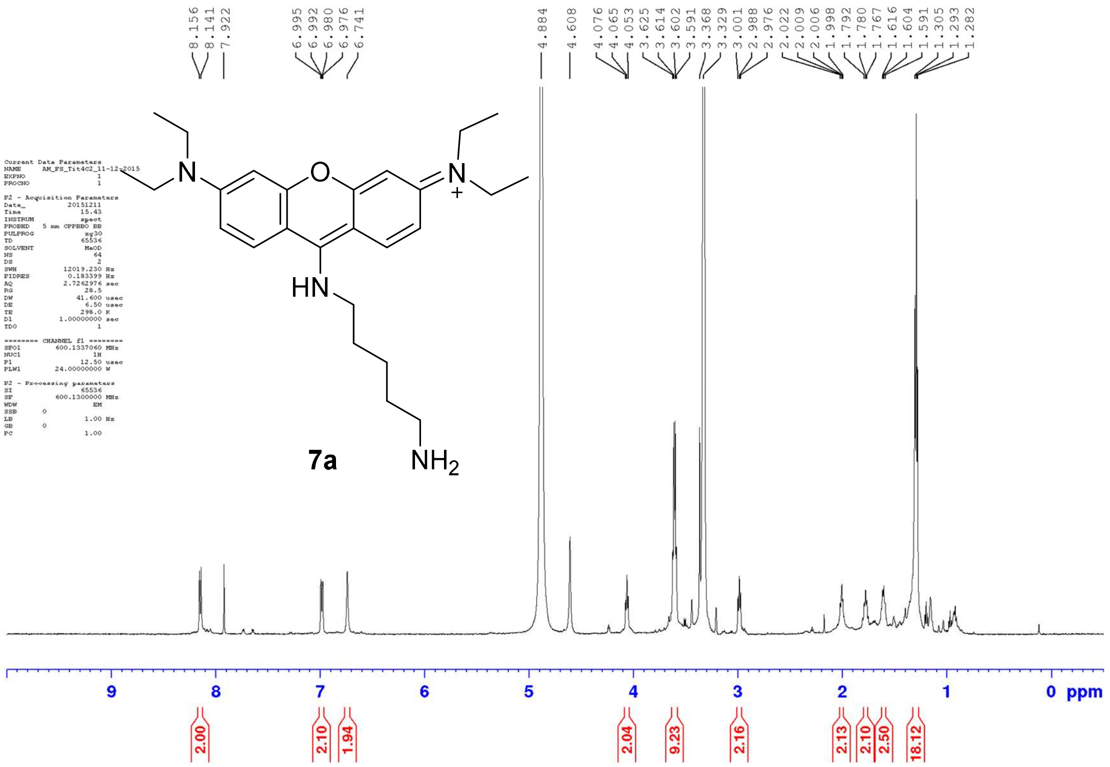

Figure S37. ^{1}H NMR spectrum (600.13 MHz, MeOD-d$_4$) of **7a**.

NMR spectrum of aminopyronin 7b

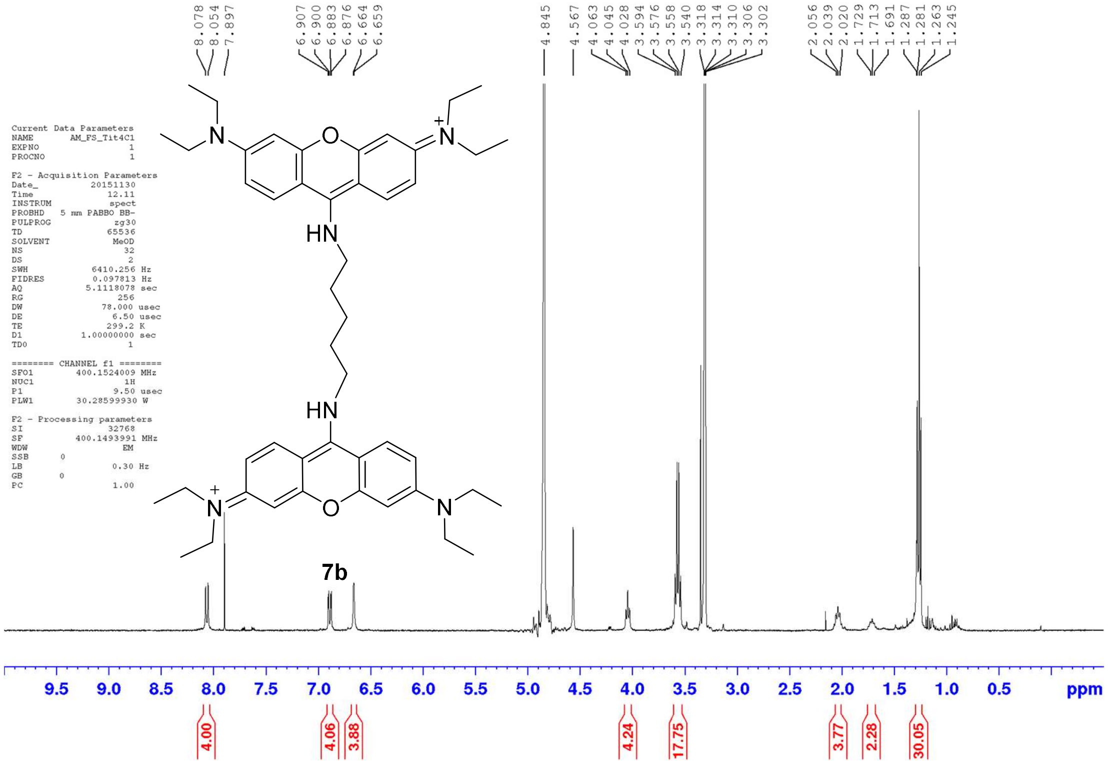

Figure S38. ^{1}H NMR spectrum (400.15 MHz, MeOD-d_4) of **7b**.

MS spectra of 5, 6a, 7a and 7b

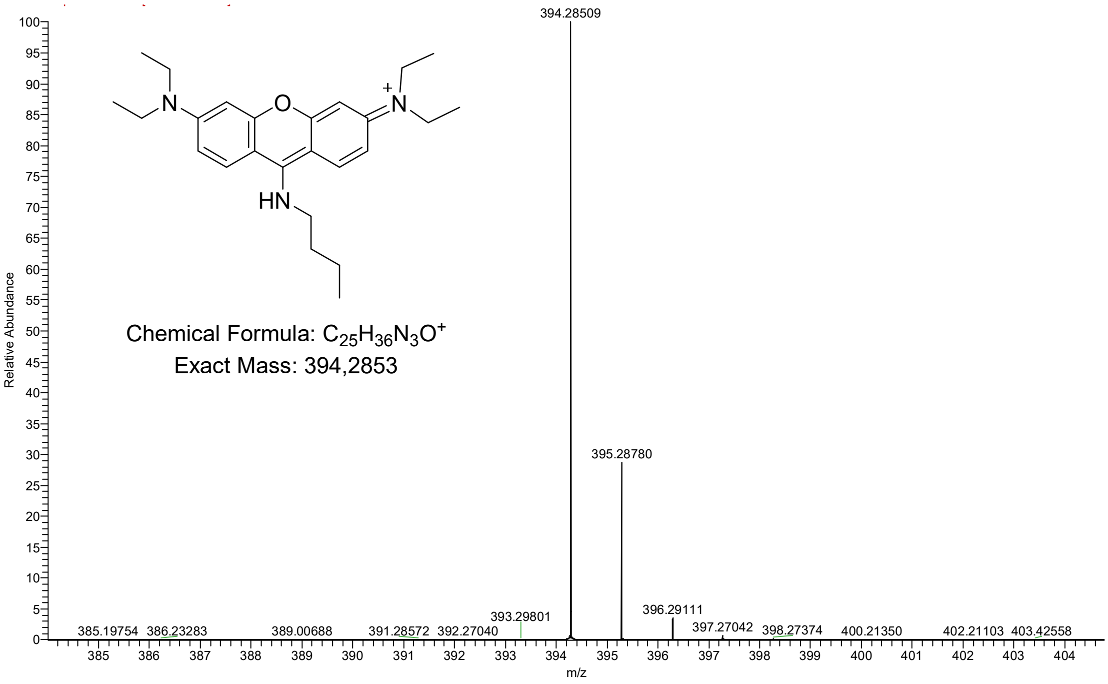

Figure S39. MS spectrum of **5**.

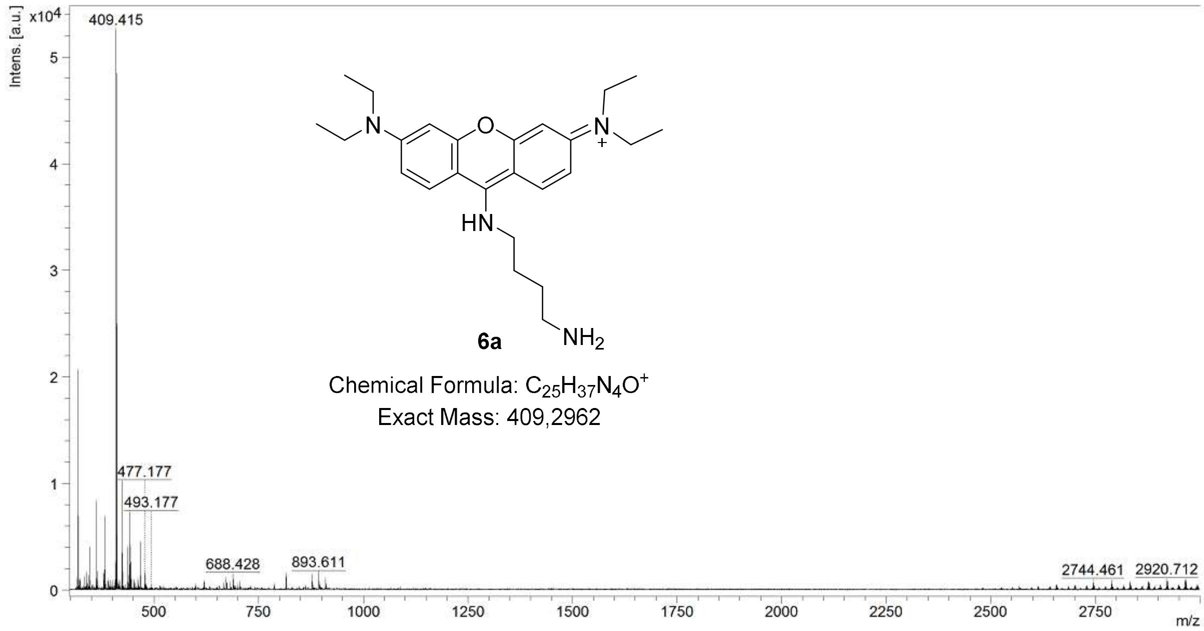

Figure S40. MS spectrum of **6a**.

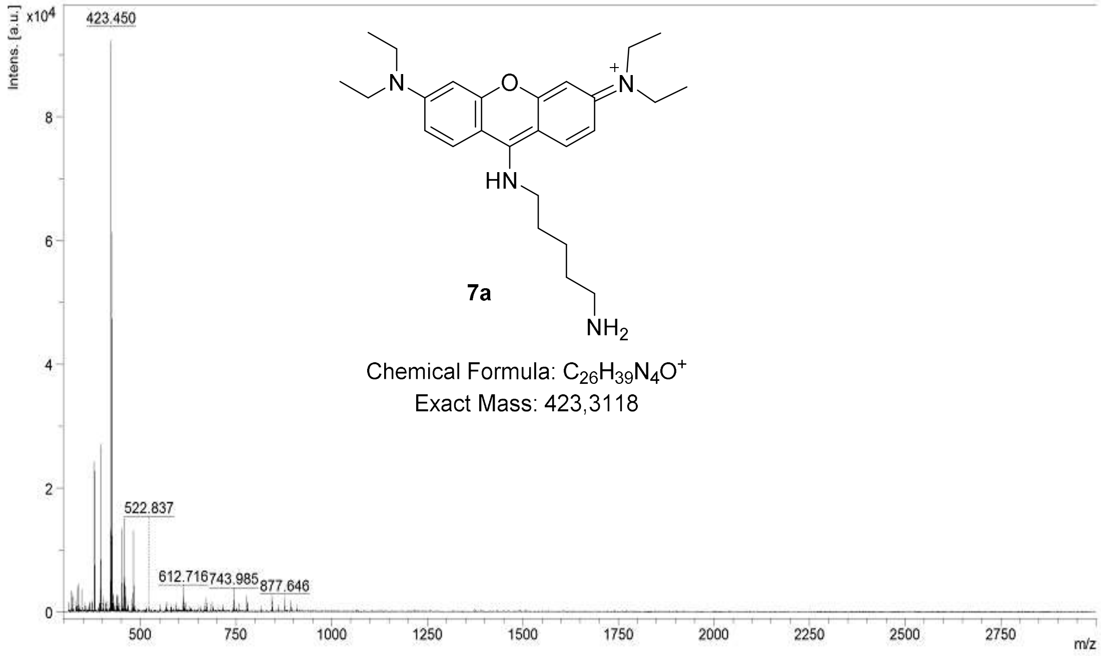

Figure S41. MS spectrum of **7a**.

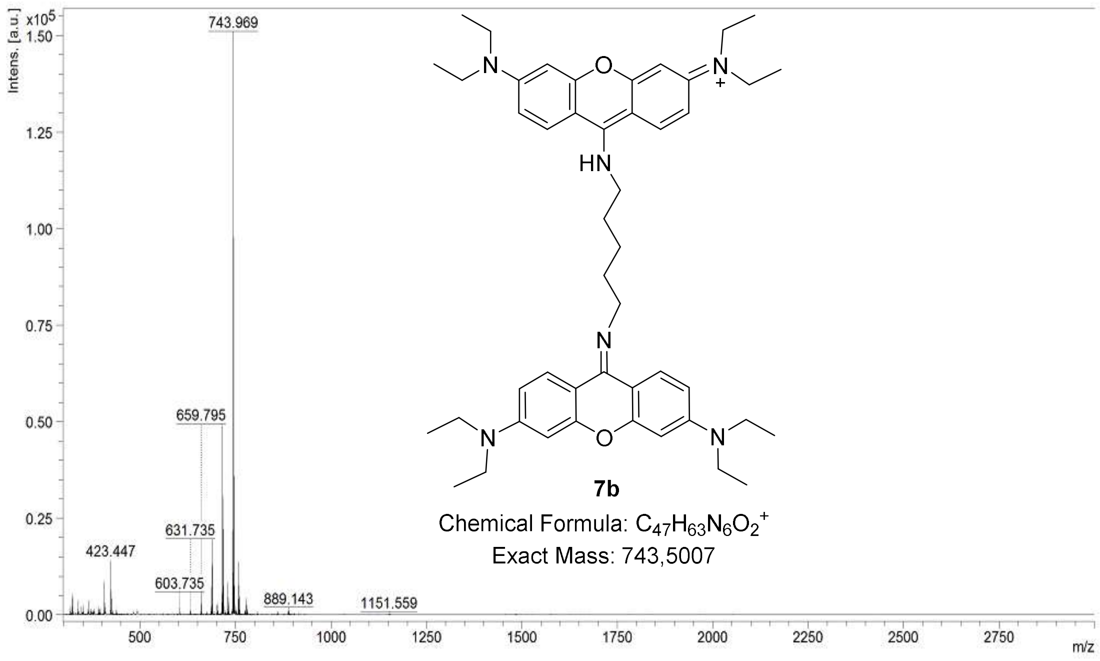

Figure S42. MS spectrum of **7b**.
